# Generation of Doubled Haploid Transgenic Wheat Lines by Microspore Transformation

**DOI:** 10.1371/journal.pone.0080155

**Published:** 2013-11-18

**Authors:** Rhoda A. T. Brew-Appiah, Nii Ankrah, Weiguo Liu, Calvin F. Konzak, Diter von Wettstein, Sachin Rustgi

**Affiliations:** 1 Department of Crop & Soil Sciences, Washington State University, Pullman, Washington, United States of America; 2 Doubled Haploid Laboratory, Pioneer Hi-Bred Int’l, Inc., Waipahu, Hawaii, United States of America; 3 School of Molecular Biosciences, Washington State University, Pullman, Washington, United States of America; 4 Center for Reproductive Biology, Washington State University, Pullman, Washington, United States of America; Nanjing Agricultural University, China

## Abstract

Microspores can be induced to develop homozygous doubled haploid plants in a single generation. In the present experiments androgenic microspores of wheat have been genetically transformed and developed into mature homozygous transgenic plants. Two different transformation techniques were investigated, one employing electroporation and the other co-cultivation with *Agrobacterium tumefaciens*. Different tissue culture and transfection conditions were tested on nine different wheat cultivars using four different constructs. A total of 19 fertile transformants in five genotypes from four market classes of common wheat were recovered by the two procedures. PCR followed by DNA sequencing of the products, Southern blot analyses and bio/histo-chemical and histological assays of the recombinant enzymes confirmed the presence of the transgenes in the T_0_ transformants and their stable inheritance in homozygous T_1∶2_ doubled haploid progenies. Several decisive factors determining the transformation and regeneration efficiency with the two procedures were determined: (i) pretreatment of immature spikes with CuSO_4_ solution (500 mg/L) at 4°C for 10 days; (ii) electroporation of plasmid DNA in enlarged microspores by a single pulse of ∼375 V; (iii) induction of microspores after transfection at 28°C in NPB-99 medium and regeneration at 26°C in MMS5 medium; (iv) co-cultivation with *Agrobacterium* AGL-1 cells for transfer of plasmid T-DNA into microspores at day 0 for <24 hours; and (v) elimination of AGL-1 cells after co-cultivation with timentin (200–400 mg/L).

## Introduction

Genetic transformation of scutellar calli in wheat by particle bombardment or co-cultivation with *Agrobacterium tumefaciens* and their regeneration into hemizygous transgenic plants is routine [1]–[3]. These procedures have however several disadvantages: A major disadvantage is lack of an efficient and reliable regeneration system after transformation of the morphogenic tissue with the gene of interest. Thus it requires preparation of large number of calli. Another disadvantage is time and labor required to confirm trans/cis-gene integration(s), genetic and molecular characterization of candidate transformants to obtain homozygous transgenic plants in the desired genetic background [4]. Collectively these procedures take a few years to get genetically true-breeding lines of selected transformants suitable for commercial applications. Additionally only a limited number of genotypes can be transformed with a reasonably high efficiency [5].

The microspores, i.e. immature pollen grains, constituting a synchronous mass of haploid cells with morphogenic potential, attracted attention of biotechnologists as a source for doubled haploid and/or transgenic plant production [6]. Based on the developmental stage targeted for transformation these procedures can be broadly classified into two groups: gametophytic and sporophytic [7]. The gametophytic route includes: (i) mature pollen-based transformations where foreign DNA is delivered into the pollen grains before pollination or applied to stigma before/after pollination and (ii) microspore maturation based transformation where foreign DNA is delivered into microspores, cultured *in vitro* into mature pollen and used for pollination to obtain transformants. The sporophytic route includes microspore embryogenesis based transformation, procedures where microspores are induced or reprogrammed towards the sporophytic pathway to produce gametic-embryos. The transformations could be performed by electroporation of androgenic microspores with foreign DNA or by co-cultivation of androgenic microspores or microspore-derived embryos with *A. tumefaciens* [8]–[16]. All of the above listed procedures have their own advantages and disadvantages, where the latter approaches have an obvious advantage as they allow production of homozygous doubled haploid transgenic plants in one generation after diploidization or by spontaneous doubling.

The rate of spontaneous chromosome doubling in bread wheat is highly variable and genotype dependent. An initial estimate by Navarro-Alvarez et al. [17] using microspore-derived wheat plants suggested a low rate (15–25%) of spontaneous doubling. Later Stober and Hess [18] reported 15–44% spontaneous doubling in German spring wheat cultivars, and Barnabás [19] reported 25–68% spontaneous doubling in winter wheat cultivars from the Central and Eastern Europe.

In view of the potential application of these approaches efforts were made to check the possibility of using electroporation to reversibly permeabilize barley microspores for foreign DNA incorporation using fluorescing propidium iodide as indicator. The initial experiments in barley clearly showed that androgenic microspores can be permeabilized by electroporation but retain their regenerative capacity under suitable conditions [20]. This has paved the way for the later experiments conducted in maize where microspores were electroporated with vectors expressing chloramphenicol acetyl transferase (*Cat*) [21] or β-glucuronidase reporter gene (*uidA*) [22]. Subsequently Obert et al. [23] conducted experiments with *uidA* to optimize the electroporation conditions for large amount of β-glucuronidase (GUS) expression, but stable transformants were not obtained in any of the above studies.

Previous investigations have shown that in addition to microspore and anther culture haploid plants can be obtained by pollination of wheat with alien species (*Hordeum bulbosum*, maize or sorghum) [24]–[26]. However, large numbers of haploid plants in a shorter duration of time can only be obtained by microspore or anther culture [27]–[33]. Although wheat doubled haploids were successfully produced as early as in the 1990s, the initial efforts to transform wheat microspores using micro-projectile bombardment resulted in only transient expression of marker genes [13], [34]. Subsequently transformation of mature wheat pollen with pDPG165 expressing the *bialaphos resistance* (*Bar*) gene under the control of the *35S* promoter by electroporation followed by vacuum drying and pollination of receptive stigma resulted in some stable transformants [35]. In a recent effort of co-cultivating anther culture derived haploid wheat embryos with *A. tumefaciens* resulted in stable doubled-haploid wheat transformants expressing the barley *Hordeum vulgare aleurone 1* (*HVA1*) gene [16].

In view of the importance of stable doubled haploid transformants in plant science, genetics and agriculture, the main quest of this communication is to develop standard procedure(s) for microspore embryogenesis based transformation in wheat. The basis of obtaining doubled haploid transgenic wheat plants is rooted in an efficient genotype independent doubled-haploid production method [36]. The procedure relies on the use of specific pretreatment, induction and regeneration media to trigger microspore embryogenesis that ensures delivery of green plants in high frequencies and reduces formation of the albino regenerants [37], [38]. Use of nursing ovaries to increase the frequency of microspore embryogenesis was also recommended in this procedure [39]. In the present communication the possibility of transforming uninucleate microspores by electroporation and co-cultivation with *A. tumefaciens* followed by regeneration of transformed microspores into doubled haploid transgenic plants was evaluated. For this purpose purified microspores were electroporated with the desired plasmid at day 0 of microspore isolation or co-cultivation with *Agrobacterium* at the same day for less than 24 h. Special procedures for killing the *Agrobacterium* cells and selection of transformed developing embryoids are presented.

## Materials and Methods

### Microspore Electroporation Based Transformation Procedure

**Figure 1:**
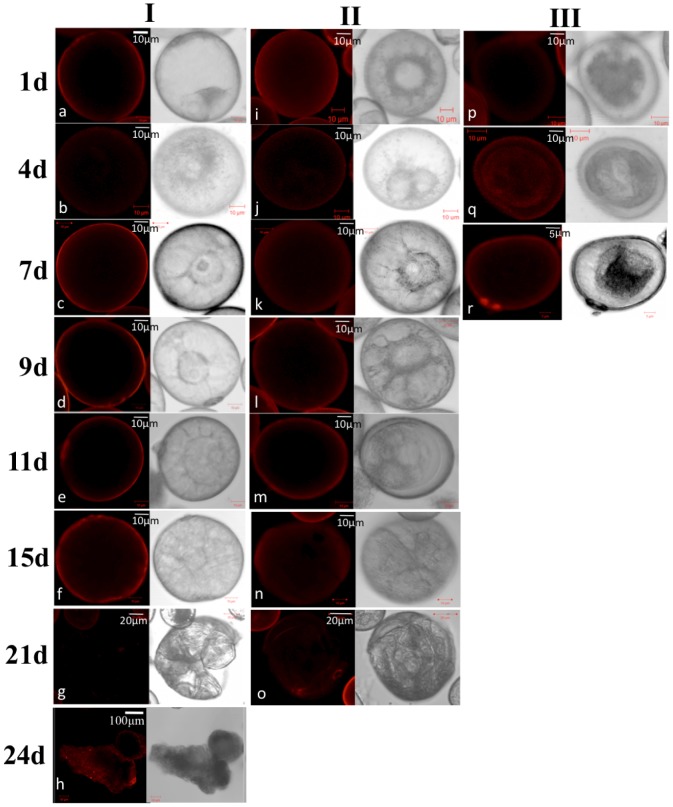
Developmental pathways of pre-treated wheat microspores in culture, as determined by time-lapse tracking. According to the type of development, microspores are grouped into three classes: type I (A–H), type II (I–O) and type III (P–R). Microspores were stained with FM 4–64 (Molecular Probes Cat. # T-3166) to confirm their viability in culture. All pictures were taken at a 25× magnification and 6× optical zoom except for G, O and H, where the former two pictures were taken at the same magnification but at 3× optical zoom and the latter picture was taken at 10× magnification and 1.7× optical zoom.

Plants of seven different wheat cultivars (Express, Chris, Perigee, Hollis, WB926, Farnum, and Louise) belonging to three market classes in the US were cultivated axenically in glasshouse maintained at 20–23°C day and 14–16°C night temperatures under a 18h photoperiod. Primary tillers at Feeke's stage 10–10.1 were harvested (below the second node from the top) (see figure S1). Appropriate tillers with microspores containing a single haploid nucleus were selected. The boots were pretreated with CuSO_4_ solution (500 mg/L, 2 mM) for 10–14 days at 4°C, a procedure, which increases the possibility of getting green seedlings. After pretreatment the spikes were sterilized for 10 min with 10% commercial bleach solution (active ingredient 6.15% sodium hypochlorite). The sterilized spikes were suspended in 50 ml of 0.4 M mannitol and blended for 10 sec at 2200 rpm in a Warring blender. The obtained slurry was sifted through four layers of cheesecloth and twice through a 100-micron mesh. The microspores are suspended in 2 ml of 0.4 M mannitol and layered over 10 ml of a 21% maltose solution. Density gradient centrifugation at 118 g for 3 min separates the non-embryogenic microspores from the embryogenic microspores, the latter accumulating at the interphase between the maltose and the mannitol solutions. The development of the isolated microspores is followed microscopically. Three cell types are easily distinguishable under the microscope i) the microspores displaying a thin intine layer and an undifferentiated cytoplasm (embryogenic), ii) the cell type showing a thick intine layer and a starch-rich cytoplasm (similar to developing pollen grains) and iii) the cell type showing an intermediate phenotype (cf. figure 1). Samples with mostly the latter cell types are discarded. For electroporation microspores are pelleted and resuspended in 0.5 ml of electroporation buffer [0.4 M mannitol + acetosyringone (98.1 mg/L)] supplemented with plasmid DNA at a final concentration of 10 ng/µL. The mixture of microspores and plasmid was electroporated at different voltages ranging from 150–1000 V using the Bio-Rad Gene Pulser. After electroporation, microspores were plated on 3ml of NPB-99 medium (table S1) in 60×15 mm Petri dishes at a minimum density of 1×10^4^/ml and a maximum density of 1×10^7^/ml. Microspores were co-cultured with living mature ovaries (3 ovaries per ml) at 28°C for embryoid development. The optimal density of microspores plated per Petri dish and the number of live ovaries used to condition culture media was identified by extensive experimentation [37]–[39]. Use of appropriate number of ovaries in the culture media is vital for getting optimal embryoid yields. The live ovaries were derived from the same explants as microspores by sampling boots in mid-uninucleate to early binucleate stage. At this stage the spikes were still fully covered by the boots. Sampled boots were surface sterilized with 80% ethanol and wrapped with one to two sheets of Kimwipes and allowed to dry off for 20–25 min. The spikes were then taken out of the boots and the ovaries were picked out of each floret with a pair of fine forceps, and added to the culture media.

Upon attaining a size of ∼2 mm about 20 embryoids per 100 mm Petri dish were transferred to the regeneration medium (per L: 0.4 mg vitamin C; 0.5 mg nicotinic acid; 0.5 mg pyridoxine; 2.5 mg CuSO_4_.5H_2_O; 0.2 mg phenylacetic acid and 0.5 mg kinetin) (table S2). After germination of the embryoids to green plantlets, the latter were transferred to Magenta boxes containing the regeneration medium followed by their transfer to pots containing potting mixture, and propagated in the growth cabinet. To avoid production of aneuploids an antimitotic drug colchicine was applied to plants at one to two tiller stages. For drug application roots were washed to remove all soil particles, and both roots and shoots were trimmed to expose meristematic tissues. After trimming roots the remaining tissues were submerged (up to the crown) in 0.1% colchicine solution bubbled with air (100 mg/L colchicine supplemented with 2% DMSO) for 7 h followed by washing in running water for another 20 h. After colchicine treatment plants were transferred to the soil.

Note: Colchicine is a natural plant alkaloid derived from *Colchicum autumnale*. It is a highly potent anti-mitotic drug. At concentrations of 0.1–1.0 µg/ml it inhibits microtubule polymerization by binding to tubulin in eukaryotic cells, which is a major constituent of the microtubules. Therefore it is commonly known as “mitotic poison” or “spindle poison”. Due to its high biological-activity at low concentrations it also functions as a potent mutagen, and causes high toxicity on ingestion and/or inhalation. On contact it may causes serious eye damage or may lead to genetic defects on ingestion. It may cause respiratory tract irritation if inhaled, and may cause skin irritation if absorbed. Thus, when working with Colchicine, it is highly recommended to stick with the safety rules about its handling and disposal.

### Microspore Transformation by Co-cultivation with *A. tumefaciens*

The following spring wheat cultivars, Chris, WED 202-16-2, and NPBCT served as source for microspores in this procedure. Microspore isolation procedure was same as earlier described for microspore-electroporation based transformation method except for the pretreatment conditions. Tillers with mid- to late-uninuclear microspores were placed below the second node from the top in a flask containing a solution with maltose, 2-hydroxynicotinic acid, 6-benzylaminopurine, 2,4D and gibberellic acid [36], and incubated at 33°C for 48–72 h. For transfection the isolated microspores (1×10^4^ microspores mL^-1^) were cultured in Petri dishes containing NPB-99 medium (table S1) supplemented with 3 live ovaries per ml of culture medium and 0.1 to 50% of recombinant *Agrobacterium* containing solution depending upon the treatment. Plasmid RS 128/Xyl (figure S2) was used for transformation, since it has been previously employed for barley transformations [40] and is expected to function similarly in wheat endosperm. RS 128/Xyl is a double cassette vector containing the *bar* selection marker between one set of T-DNA left and right borders and the codon-optimized target gene for 1,4-β-xylanase of *Bacillus subtilis* (GenBank accession no. AY902462) between the second set of T-DNA borders. The target gene was driven by the D-hordein gene *hor3-1* promoter and was supplied with the code for the signal peptide launching the newly synthesized enzyme precursor into the pathway for endosperm protein storage [41]. The nucleotide sequence of the *hor3-1* promoter is 87% identical to the high molecular weight glutenin subunit promoter of wheat [42]. Plasmid RS 128/Xyl was transferred into the disarmed Ti plasmid of *Agrobacterium*-strain AGL-1 by electroporation [43] resulting in strain MT1. Transformation of the purified microspores by co-cultivation of androgenic microspores with MT1 was carried out for 1–48 h with various concentrations (0.1 to 50%) of MT1 in the culture media at days 0, 1, 3, 5, 7, 14, 21 and 30. Filtration, rinsing and timentin in the induction medium (NPB-99) were tested for elimination of *A. tumefaciens* MT1 after a desired period of co-cultivation. As *A. tumefaciens* cells are smaller than microspores (<5 vs 40- µm), a filter with a mesh pore size of 38 µm was constructed and the co-cultivation medium poured through the filter. The retained microspores were rinsed, collected and returned to the liquid embryoid induction medium. Timentin at 0–800 mg·L^-1^ concentrations was tested to determine optimal concentration(s) to kill escaped MT1 cells. Fresh ovaries were then added to the plate and incubation carried on in the dark at 25°C. Mature embryoids of 1–2 mm formed in 4–6 weeks, at which time they were aseptically transferred to solid bialaphos-containing 190-2 medium (see table S1) in 100×15 mm Petri dishes for plant regeneration. The embryoids were incubated under continuous fluorescent light at 22°C. In approximately 2 weeks green plants developed and were transferred subsequently to soil and grown to maturity.

### Molecular Characterization of MT1 (128/Xyl) Transformants

Selection of transformants was carried out at the embryoid germination stage. DNA was extracted according to Horvath et al. [43] and primary transformants were identified by PCR using primers Bar5 (5′-CGGCGGTCTGCACCATCGTCAACCAC-3′) and Bar3 (5′-GGCATATCCGAGCGCCTCGTGCATG-3′) amplifying 373 bp from position 56 at the 5′ end of the *bar* gene and position 428 at the 3′ end of the gene. Primer pair Hor5 (5′-AAGCTTCGAGTGCCCGCCGATTTG-3′) and Liuxyldown (5′GTA GCGCGTGGTCGTGTAGATGTCG-3′) amplified 837 bp from position 39 at the 5′ end of the D-hordein gene promoter and position 875 at the 3′ end of the *xylanase* gene. PCR for the *xylanase* gene was carried out in 25 µl reaction volume containing 50 ng of genomic DNA, 1.2 pmol of each primer, 0.2 mM dNTPs, *Pfu* buffer [10 mM KCl, 10 mM (NH_4_)_2_SO_4_, 20 mM Tris-HCl (pH 8.75), 2 mM MgSO_4_, 0.1% Triton X-100, and 100 µg/ml BSA] and 1 U of *Pfu* polymerase. Amplification was performed in a PTC-100 programmable thermal controller (MJ Research, Cambridge, MA) at 97°C for 1 min, annealing at 58°C for 20 sec and extension at 72°C for 30 sec followed by 29 cycles of 97°C for 30 sec, 60°C for 20 sec and 72°C for 1 min; this was followed by 5 min at 72°C. PCR reactions for the *bar* gene were also carried out in 25 µl reaction volume containing 50 ng of genomic DNA, 0.8 pmol of each primer, 0.2 mM dNTPs, *Taq* buffer (20 mM Tris-HCl, pH8.4, 50 mM KCl), 10% DMSO (vv^-1^), and 1 U of *Taq* DNA polymerase. DNA was denatured at 94°C for 3 min, followed by 25 amplification cycles of 94°C for 45 sec, 58°C for 30 sec and 72°C for 1 min; this was followed by 5 min at 72°C.

To confirm that the DNA template in the PCR reactions for the *bar* gene was not from the plasmid DNA due to potential *Agrobacterium* contamination on the leaves, primer set Bar-Ubi1-up (5′-CTTTCCCCAACCTCGTGTT-3′) and Bar-Ubi1-down (5′-GTACGGAAGTTGACCGTGCT-3′) was designed. This primer set amplified a 1212 bp fragment with plasmid DNA as template (positions 2869 to 4080). However, this same primer set would amplify a 198 bp fragment with the transformant cDNA as template due to the intron removal (positions 2954 to 3967). The cDNA was obtained by RT-PCR using total RNA isolated from transformants with TRIzol reagent following the procedure described in Lee et al. [44]. RT-PCR product containing the cDNAs was used in PCR reactions for the *bar* gene. The reactions were carried out in a total of 50 µl reaction volume, consisting of 1 µl of cDNA from RT-PCR, 1 pmol each of Bar-Ubi1-up and Bar-Ubi1-down primers, 0.15 mM dNTPs, 1 x Red *Taq* buffer, 0.25 mM MgCl_2_ and Red *Taq* Polymerase (Sigma D5684). DNA was denatured at 95°C for 4 minutes, followed by 35 amplification cycles of 95°C for 1 min, 58°C for 1 min, and 72°C for 1 min followed by final extension at 72°C for 5 min. After PCR, 40 µl PCR mixture was directly used for 1% agarose gel electrophoresis using 1 x TAE buffer.

### PCR Based Confirmation of Microspore Electroporation Based Transformants

After four weeks on rooting media, the plants were kept for another 4 weeks on soil before collecting leaf samples for DNA extraction. DNA was extracted using Biosprint Plant DNA extraction kit (Qiagen, Valencia, CA) following manufacturer's instructions. Candidate transformants were tested by PCR using gene-specific primers to confirm transgene integration(s) (for PCR conditions, see table S3). Bands of expected sizes were excised from the agarose gel and DNA was eluted from the bands using Geneclean kit following manufacturer's instructions (MP Biomedicals). The eluted DNA was used as template for the sequencing reaction using either forward or reverse primers in separate reactions. Sequencing reactions were carried out in 10 µl reaction mixtures, each containing 200 ng template DNA, 0.35 µM of either forward or reverse primers, and BigDye® mixture (Applied Biosystems) using the following PCR profile: 96°C for 10 sec, 50°C for 15 sec, 60°C for 6 min with 24 iterations. Nucleotide sequences were edited using the DNAStar (Lasergene Software).

### Southern Blot Hybridization

The methods used for DNA isolation, Southern blotting and hybridization were as described in Kleinhofs et al. [45] and Horvath et al. [43]. Ten µg of *Hin*dIII-digested genomic DNA was separated by agarose gel electrophoresis, blotted onto nylon membranes, and hybridized with the [-^32^P]dCTP-labled coding region and promoter as well as signal peptide sequences of the transgene xylanase (837 bp) using the All-in-One random labeling system (Sigma R7522, R9647).

### Quantitative Determination of Endochitinase Activity in Plant Tissues

Tissues were ground after adding 400 µL of extraction buffer (50 mM Na-acetate with 100 µg/mL BSA, pH 5.5), vortexed, and centrifuged for 10 min at 18,000 x g. The supernatant was transferred into a fresh tube and stored at 4°C. For the quantitative assay, 5 µL of protein solution were mixed with 45 µL Na-acetate buffer. Five microliters of 1∶20 diluted sample were mixed with 45 µL Na-acetate buffer and 0.5 µg methylumbelliferyl-chitotrioside (Sigma). Samples were incubated at room temperature for 10 min with gentle shaking. Fifty µL of 0.3 M glycine/NaOH buffer (pH 10.6) were added to stop the reaction. Fluorescence was measured using a Safire spectrophotometer (Tecan) (excitation/emission 455 nm/360 nm). The amount of enzyme was determined with the help of a standard curve prepared using ThEn-42 enzyme expressed in *Pichia pastoris*. Each assay was replicated thrice.

### Enzymatic Assay for Xylanase Activity

Transgenic xylanase in the transformed grain was measured in two ways: (i) For the zymogram method [46] the wheat grains were cut into half, and placed onto plates with the cut end facing down. The plates contained 3% (wv^-1^) oat-spelt xylan (Sigma, St. Luis, MO) in 0.05 M glycine buffer, pH 6.0 and 1% agarose (wv^-1^). After overnight incubation at 50°C the plates were stained in 0.1% Congo Red for 15 min. Congo Red stains xylans, but half grains from which xylanase has diffused into the medium show an unstained ring on the red background while untransformed grains will lack the unstained ring. (ii) To measure the amount of xylanase 200 mg of ground powder of wheat grains was dissolved in 0.7 ml 0.05 M glycine buffer pH 6.0. The enzyme extract was collected by centrifugation at 17,949 g for 10 min. 150 µl of the enzyme solution was mixed with 200 µl of azo-birchwood xylan Brilliant Blue R (Megazyme, Co. Wicklow, Ireland) and incubated at 50°C for 30 min. The reaction was terminated by adding 1 ml precipitant from Megazyme kit (containing Na-acetate, Zn-acetate, HCl and 2-methoxyethanol). Unhydrolyzed azo-xylan was removed by centrifugation. The supernatant was transferred into a clean tube and the optical density of the water-soluble products released from the azo-xylan measured at A_590_. The amount of xylanase was calculated with a standard curve made with purified xylanase enzyme.

### GUS Histochemical Assay

Expression of the GUS in transgenic plants carrying construct pRB107 (figure S2A) was assayed by using 5-bromo-4-chloro-3-β-D-glucuronide as substrate using a modification of the procedure described by McCabe et al. [47]. An inch long leaf and root fragments from the transformed and untransformed plants (control) were stained by immersing the tissue in GUS staining buffer followed by incubation at 37°C overnight. Chlorophyll was extracted by consecutive 24-h washes with 25%, 50%, 75%, and 95% ethanol. To study GUS expression in mature grains from transformants their progeny and control (wheat cv. Louise in this case) three water imbibed (after 24 h) and 3 dry grains from each genotype were divided in two equal halves and immersed in GUS staining buffer followed by incubation at 37°C for 19 h. After staining and washing the tissue was examined under a SMZ800 Nikon dissecting microscope equipped with a camera.

### Mitotic and Meiotic Chromosome Analysis

Root tips from germinated seeds were excised and pretreated in chilled water for 20-22 hours, fixed in ethanol:acetic acid (3∶1, v∶v) solution for a week at room temperature and stored at 4°C till analysis. The material was stained in acetocarmine solution for 1 hour (RT) and incubated at 100°C for a minute and squashed in 45% acetic acid. Mitotic cells were analyzed with a Leica DMLB microscope equipped with a Cohu CCD camera and the Leica QWin software. Twenty cells per genotype were examined for their chromosome complements. For meiotic analysis the immature spikes were fixed in Carnoy's solution (ethanol 60%/chloroform 30%/acetic acid 10%) and analyzed using the standard procedure described in Sharma and Sharma [48]. Ten cells per genotype were examined for the number of bivalents.

### Confocal Microscopy

GFP activity was monitored in pUbi.GFP transformed plants using a Bio-Rad MRC 1024 laser scanning confocal microscope with a 25-mW krypton/argon laser (Bio-Rad, Thornwood, NY, USA) adjusted at 488 nm excitation and 510 nm emission. The T_1_ ovaries and T_2_ root were sectioned using Leica cryotome and mounted in water under cover slip for visualization under microscope. Images were acquired using the LaserSharp2000 software (Bio-Rad, Thornwood, NY, USA).

### Transmission Electron Microscopy

Enlarged microspores after pretreatment were assayed for ultrastructural details. To obtain electron micrographs samples were prepared following Maraschin et al. [49] and the sections were observed using a Jeol 1200 Ex electron microscope at the WSU Franceschi Microscopy and Imaging Center.

## Results and Discussion

### Time-lapse Tracking and Ultra-structural Study of Wheat Microspores

**Figure 2:**
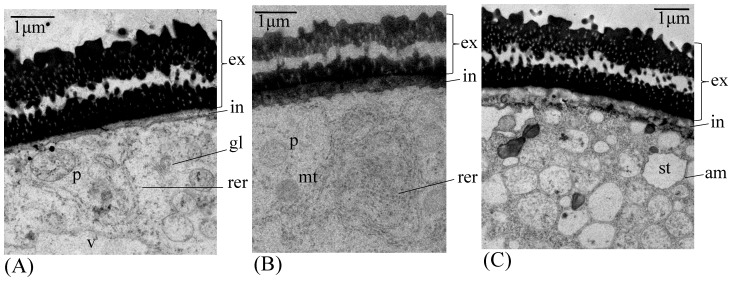
Electron micrographs of wheat microspores after pretreatment using transmission electron microscopy. Figures a-c show differences in thickness of intine, number of cytoplasmic organelles and amount of starch accumulated in amyloplasts. (A) represents type I developmental pathway, (B) represents type II developmental pathway, and (C) represents type III developmental pathway. am =  amyloplast, ex =  exine wall, in =  intine layer, mt =  mitochondria, gl =  Golgi apparatus, p =  proplastid, rer =  rough endoplasmic reticulum, st =  starch.

Similar to barley, time-lapse tracking of wheat microspores clearly showed three developmental pathways for microspore development. The fate of developing microspores depends upon their responses to the pre-treatment conditions, which direct them to follow different developmental pathways (figure 1). These cellular pathways have their hallmarks, which allow differentiation of the microspores during their development in the cultures. For instance, the ultra-structural differentiation of microspores analyzed by transmission electron microscopy revealed three cell types (figure 2), where the first type displayed a thin intine layer and an undifferentiated cytoplasm (figure 2A and figure S3A in higher resolution), the third type showed a thick intine layer and a starch-rich cytoplasm (similar to *in planta* developing pollen grains; figure 2C and figure S3C) and the second type showed an intermediate phenotype (figure 2B and figure S3B). Accumulation of starch in the pollen amyloplasts marks the commitment to the pollen developmental pathway [50]. Our observations indicate that the microspores that attain pollen morphology even after pretreatment with specific conditions, are still committed to the gametophytic pathway, and represent the type III developmental pathway as identified by time-lapse tracking study in barley [49]. On the other hand, microspores with undifferentiated cytoplasm indicate repression of the gametophytic pathway. Prior to induction of androgenesis, wheat and barley uninucleate microspores are characterized by the lack of differentiated cytoplasmic organelles and presence of a thin intine layer. This suggests that, in wheat and barley, the microspores after different treatments, with few differentiated cytoplasmic organelles and a thin intine layer signifies repression of the gametophytic pathway (i.e. type I and type II microspores; figure 2A, B) [49], [51], [52]. The maintenance of a thin intine layer represents an early morphological marker for androgenic microspores in wheat. Tracking showed that the first developmental change associated with dividing microspores (type I and type II) was a star-like morphology, which was characterized as a transitory stage between vacuolated microspores after stress treatment and the initiation of cell division (figure 1C) [50]. Although the star-like morphology appears to be a morphological marker for the initiation of cell division in stressed microspores, a star-like morphology *per se* does not assure that a microspore will ultimately follow the embryogenic pathway. The occurrence of a star-like morphology is a dynamic process, in which the time of occurrence will depend on the type of stress applied and the stage of microspore development [50]. In wheat, type I microspores display the tendency to acquire star-like morphology later than type II microspores.

The morphological markers identified from the time-lapse and ultrastructural studies provided the efficiency to identify wheat microspore culture at an early stage, which can give rise to large number of viable calli and in turn has higher scope of producing candidate transformants after transfection.

### Factors Affecting Microspore Transformation and Their Regeneration into Green Plants

To determine optimal conditions for microspore electroporation-based transformation we transformed wheat microspores with the following expression vectors: pUbi.GFP, pRB107 and pRB113, respectively carrying green fluorescence protein (GFP), β-glucuronidase (GUS) and codon optimized endochitinase gene from *Trichoderma harzianum* (figure S2). A variety of transfection and culture conditions were tested (figure S4), which includes a wide range of electroporation voltages from 150-1000 V. A value of ∼375 V produced maximum number of transformants. A pretreatment of immature spikes with CuSO_4_ solution (500 mg/L) at 4°C for 10 days and incubation of microspores after transfection sequentially at 28°C in NPB-99 medium and at 26°C in MMS5 medium (tables S1 & S2) resulted in recovery of large numbers green regenerants (figure S4C). Application of copper sulphate during pretreatment has shown to decrease the number of albinos in wheat as previously shown in barley, but the exact mechanism underlying the beneficial effect of copper is not understood [53].

Using these conditions, transformations were performed with microspores from the wheat cultivars Express, Chris, Perigee, Louise, Farnum, Hollis and WB926 (cf. Material and Methods & table S4). A great variation in the performance of different wheat cultivars in terms of their androgenic potential and number of recovered green regenerants was observed. Express and Perigee showed highest androgenic potential, whereas Hollis and WB926 gave highest number of green regenerants (table S4).

Similarly conditions were optimized for microspore-based Agrobacterium mediated transformation procedure. Isolated and purified microspores of the wheat cultivars Chris, WED 202-16-2 and NPBCT were co-cultivated with A. tumefaciens strain MT1 cells derived from A. tumefaciens strain AGL-1 cells by electroporation with plasmid RS 128/Xyl (figure S2D) (cf. Material and Methods). Both microspore viability and embryoid production decreased with increased numbers of MT1 cells in the co-cultivation medium (table S5). When solution containing 20% MT1 cells was added to the microspores the maximum permissible co-cultivation duration for obtaining embryoids and plant regeneration was 45 minutes (table S6). In further experiments it was found that inoculation concentrations ranging from 1–20% of MT1 cells for less than 5 hrs of co-cultivation or 0.1–1% MT1 cells for 24 hrs of co-cultivation were usable for microspore transformation and androgenesis with the genotypes Chris, WED 202-16-2 and NPBCT, but co-cultivation had to be carried out at day 0 immediately after purification of the microspores.

It is essential to use timentin for killing remaining *A. tumefaciens* cells after co-cultivation. Results showed that number of embryoids decreased with increasing concentration of timentin in the embryoid induction medium (table S7). The optimal concentration of timentin was determined to be 100 to 400 mg/L. With these doses of timentin present in the medium, a reasonable number of embryoids were produced while maintaining their regeneration efficiency. The time of adding timentin to the culture medium was found critical. If the AGL-1 cells were not killed post-co-cultivation by timentin, AGL-1 cells grew back rather fast, within a couple of days. If this happened, it was difficult to kill AGL-1 cells by reapplying timentin without severely inhibiting embryoid formation.

For selection of transformants the *bar* gene was used. It provides resistance to bialaphos through expression of phosphinotricin acetyl transferase. A test on wild type Chris microspores indicated that with 2 mg·L^-1^ bialaphos or higher concentrations in the 190-2 regeneration medium (table S1) only a few 1–2 mm embryoids survived and developed shoots but no roots (table S8). When small wild type embryoids that had been germinated for 7 days on 190-2 plant regeneration medium were transferred to 4 mg·L^-1^ bialaphos containing medium plant regeneration was completely inhibited while 38% of plant regeneration was obtained on medium containing low doses of bialaphos (table S9). Seventy percent of these germinating embryoids survived, when transferred to media with the required high dose of bialaphos (table S9). In view of these observations we performed a screen with the wild type Chris embryoids using increasing concentrations of bialaphos in 190-2 medium to determine a critical concentration of the herbicide to start the staggered selection. Results of the analysis suggested 2 mg·L^-1^ bialaphos to be an appropriate starting point for the staggered selection for Chris (table S8). As similar information does not exist for WED202-16-2 a higher concentration of 4 mg·L^-1^ bialaphos was used directly. When these selection schemes were applied to the transformed microspores (table S10) they permitted to obtain 38 candidate transformants.

### Molecular Characterization of Microspore Electroporation Based Transformants

Around 30,000 enlarged microspores were electroporated with each construct (involving 6 electroporation events including ∼5,000 cell each in ½ ml electroporation buffer) and transferred to 60×15 Petri dishes. As observed in the time tracking study close to 94% of the enlarged microspores followed the gametophytic pathway (type III) or ceased growth after few cell divisions forming a globular mass of cells (type II), leaving a small proportion of cells that developed into embryo like structures (ELSs). The number of wheat microspores that develop into ELSs corresponded well with the previous reports in barley [49], [54], but deviated significantly from what was reported for an Austrian winter wheat cv. Ferdinand by Indrianto et al. [52]. In this study Indrianto and co-workers reported development of 89 (12.16%) of 732 microspores in embryo-like structures, which is double the number of ELSs observed in the present study. The deviation in number lies in the way one defines ELSs, which in our case are multicellular structures released out of the microspore wall (including exine and intine) instead of all multicellular/globular structures (figure 1G, H). Out of ∼1,800 ELSs observed per construct only 0.33, 0.44 and 1.28% of ELSs regenerated into green plants from pUbi.GFP, pRB113, and pRB107 transformed microspores, respectively. The small number of ELSs observed in the present study is a cumulative effect of electroporation on enlarged microspores, tendency of microspore-derived embryos in wheat to regenerate into albinos [55] and the after effect of colchicine treatment [16], which is known to induce chromosomal aberrations leading to mortality. Out of these green regenerants 4 plants showed transgene integration for pRB107 in Louise, Chris and Farnum backgrounds, 4 plants showed integrations for pRB113 in Louise and Express backgrounds and 3 plant showed integrations for pUbi.GFP in Louise background. Nine of these 11 transformants produced grains including 2 plants transformed with pRB107, 4 with pRB113 and 3 with pUbi.GFP, of which 2 plants each transformed with pRB113 and pUbi.GFP produced only 2–6 grains thus these plants were not included in the further analyses. The transformation efficiency achieved (∼one transgenic plant per 8200 isolated microspores) was superior to prior androgenic microspore-culture based methods, and is comparable to the efficacy observed in the *Agrobacterium* mediated transformation of barley pollen cultures that resulted in 3.7 T_0_ plants per donor spike [8]. In the earlier studies low transformation efficiencies of either one transgenic plant per 5.5×10^6^ embryogenic pollen derived protoplasts or one transgenic plant per 10^6^–10^8^ bombarded immature pollen grains was reported [9]. Considering the ease with which >30,000 microspores can be isolated from 6–8 spikes derived from only three donor plants, and induced to form ELSs this method has a great advantage over other methods like *Agrobacterium*-based or biolistic transformations, where labor intensive and time-consuming steps of embryo culture are required.

Although we do not expect chimerism in microspore derived transformants, but to eliminate this possibility we tagged 1–14 individual spikes from each green regenerant to check for transgene integration(s), and collected leaf material (mostly a part of flag leaf) for DNA extractions (hereafter each spike was treated as an individual transformant). To our surprise, DNA samples collected from the same plant showed chimerisms with gene integration in only 1–4 samples collected per plant. In wheat the harvested embryogenic microspores turned out to be a population of uni/bi-nucleate microspores with generative and vegetative nuclei that could lead to chimerism [9]. Since we have not applied any kind of selection during propagation of transfected microspores, it is possible that nascent embryonic cells formed after a few divisions, in the absence of selection pressure, propagated cells lacking the foreign DNA fragment and led to chimeric calli, but additional work is required to address this issue. The regeneration of chimeric plants after electroporation of microspores can also be explained by considering the possibility that transgene integration occurred only after initial cell divisions. But it is difficult to deduce when during regeneration the integration takes place, albeit its inheritance through generations proved that at some point, integration did occur. Most likely due to lack of the selection pressure both kinds of cells with or without transgene integration proliferated to give rise to a chimeric plant.

It has been revealed by the efforts in past that most of the transgene integrations are sequence-independent and mostly relies on illegitimate recombination events [56]. These earlier studies also suggested that the transgene integrations preferentially take place at transcriptionally active euchromatic regions of the genome that mostly reside distally on the chromosomes [57], [58]. Analyses of transformed cereals using fluorescent *in situ* hybridization suggested that the integrations in most of the cases take place within or in proximity of genes that triggers cells internal vigilance system to eradicate these integrations during the process of cell division [56], [59]–[61]. It allows cells to maintain their genomic integrity or transcriptional state by keeping epigenetic status of the integration site unaltered. As most of these integration sites are hypomethylated they also show high transposable element activity, which by their movement lead to single/double stranded DNA breaks, which activate the cell's inbuilt repair system [58]. This process could also lead to transgene eradication from integration sites resulting in chimeric plants.

According to another hypothesis a single shoot can originates from multiple cells and leaf tissues derived from such shoots are composed of several layers. Among these layers, the L2 is responsible for the generation of male and female gametes [62], if the origin of the L2 layer takes place from nontransformed cells it would result in the production of chimeric plants. The earlier observations suggested that under no selection, more L2 layers in the putative transformants originated from the nontransformed cells giving rise to chimeric organism [63].

In view of the above, to reduce the chimerism problem in the microspore electroporation based method co-transformation with vector carrying selectable marker gene or use of double cassette vectors is recommended, which is currently under testing.

**Figure 3:**
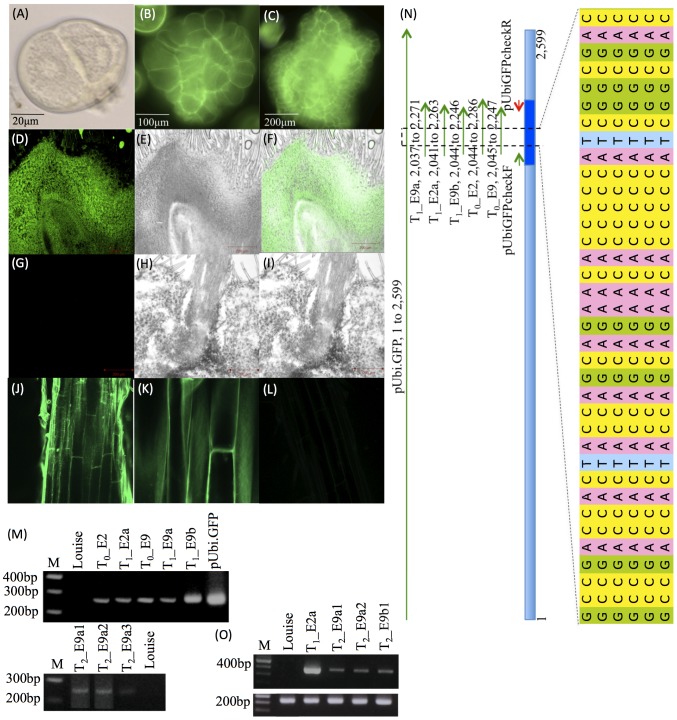
Cytological and molecular characterization of transformants with pUbi.GFP. (A) An androgenic microspore of wheat cultivar Louise. (B and C) embryoid showing GFP expressing at T_0_. (D–F) Section of T_1__E2a ovary showing GFP expression, left under GFP filter, middle white light and right an overlay of the two images. (G–I) Section of control ovary, left under GFP filter, middle white light and right an overlay of the two images. (J and K) Section of T_2__E2a3 root showing GFP expression, left at 25× and right at 63×. (L) Section of control root visualized under GFP filter. (M) PCR based confirmation of transgene integration at T_0_, T_1_ and T_2_ generations using gene specific primer pair pUbiGFPcheck (see Table S3). M = 100 bp ladder. (N) Sequence based confirmation of transgene integration. In the line diagram, pUbi.GFP represents the vector sequence, pUbiGFPcheck F and R represent the primers used to amplify *GFP* gene from the wheat genomic DNA/plasmid DNA and T_0__E2, T_0__E9, T_1__E2a, T_1__E9a and T_1__E9b represent the PCR products amplified from the genomic DNA of transformants and their progenies. A small part of the sequences was magnified to show integration of transgene in the wheat genome. (O) Confirmation of transgene expression by RT-PCR using T_1_ and T_2_ cDNAs amplified with gene-specific primer pairs GFPcheck F & R (Table S3) and Glyceraldehyde 3-phosphate dehydrogenase (GAPDH; used as control). [Note: The naming convention used to number transgenic events and their progeny is as follows. The first letter is to represent each regenerated plant. The number following this letter is to represent the tiller sampled from the regenerant. This number is followed by another letter that represents the T_1_ plant and the number succeeding this letter represents the T_2_ plant. Same naming system was followed throughout the manuscript.]

Out of four positive T_0_s for pRB107 two plants were totally sterile, and for the remaining two plants 9 and 14 spikes were collected, where 3 and 2 spikes respectively showed transgene integration(s). Four-six seeds each were propagated for the further analysis. PCR followed by sequencing of the PCR products confirmed inheritance of transgene from T_0_ to T_1_ and from T_1_ to T_2_ generations (figure S5A; for PCR conditions and primer details, see table S3). Transcript analysis of the positive individuals confirmed transgene expression. The water imbibed and dry grains derived from two respectively three positive tillers from two regenerants designated as D and C and their T_1_–T_3_ progenies were stained with GUS staining buffer. Weak to strong GUS activity was observed in the seed coats of these transformants and their progenies (figure S5A). However the histochemical assays of leave and roots of these transformants were negative (see Materials & Methods). This could be attributed to the presence of an unknown inhibitor in these wheat tissues interfering with the β-glucuronidase activity as was previously reported by Bahieldin et al. [64]. Similarly for pRB113 two transformants were studied in detail, where five out of 10 collected spikes from one plant and one spike from another plant showed presence of transgene at the T_0_ generation. When 4–6 seeds each for the positive T_0_s were propagated and tested by PCR all showed faithful inheritance of the transgene at the T_1_ and T_2_ generations (figure S5B), and also showed transgene expression at both generations by RT-PCR (figure S5B). However, variation in enzyme quantities (ranging from 1.31–57.54 ng/grain) was identified by the biochemical assay (figure S5B; cf. Materials & Methods). For pUbi.GFP also one transformant in Louise background was studied in detail: Two of the 14 collected spikes showed transgene integration, and inheritance in both T_1_ and T_2_ generations (figure 3M). Expression of transgene was also confirmed by RT-PCR (figure 3O), while gene activity as fluorescence emitted by GFP could be visualized only in one of the two cases (figure 3A–L). Reason for observed variation in the biochemical activities of different transformants is unclear at this time point. However, it can be attributed to the presence of unidentified inhibitors, low efficiency of the *35S* promoter in absence of cereal-gene derived intron in the 5′ untranslated sequence of the gene to be expressed, silencing of integrated gene(s), or gene integration at transcriptionally inactive site in the genome. But finding exact reason of the observed variation requires further investigation. As expected, no segregation of transgene was observed when 4 T_1_s were tested for each positive T_0_ (spike). The doubled haploid nature of the plants was further validated by chromosome counts at the mitotic metaphase by squashing root-tips collected from each T_1_ seedling and by counting number of bivalents at the meiotic metaphase I by squashing immature anthers excised from the florets of T_1_ spikes (figure S6; see Materials & Methods).

### Molecular Characterization of MT1 (128/Xyl) Transformants

**Figure 4:**
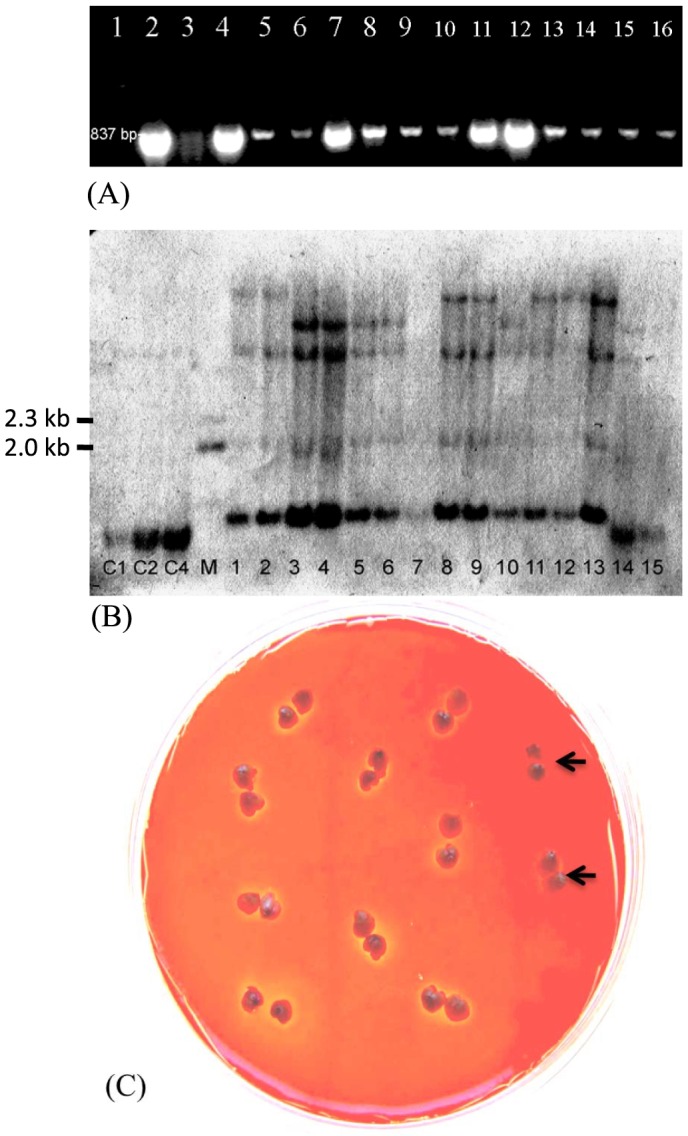
Characterization of MT1 (128/Xyl, cf. Fig. S1e) transformants. (A) PCR analysis of 13 randomly chosen doubled-haploid T_1_ seedlings of primary transformant MT1 B4. Lanes: 1 = 1 kb ladder; 2 =  plasmid DNA; 3 =  wild type wheat DNA; 4–16 = T_1_ seeds. (B) Southern blot analysis of homozygous MT1 transformants from T_1_ seedlings with the xylanase gene. Lanes: C1, C2, C4 respectively showing 2, 4 and 8 copies of 837 bp probe; M =  DNA ladder; lanes 1–10 = T_1_ doubled-haploid seedlings of 6 different T_0_ transformants (1 and 2 = MT1-1, 3 and 4 = MT1-2, 5 and 6 = MT1-3, 7 = MT1B5, 8 and 9 = MT1-6, and 10 = MT1-7); lanes 11–13 = T_1_ doubled-haploid seedlings of MT1-B4; lanes 14–15 =  wild type DNA. (C) Zymogram assay for identification of transgenic wheat grains synthesizing recombinant 1,4-β-xylanase. Transgenic wheat grains (T_2_ of MT1-B4) secrete the enzyme into the medium containing oat-spelt xylan that is stainable with Congo Red. De-polymerization of the xylan by the enzyme results in an unstained yellow ring around the seed. Wild type wheat grains lack the yellow ring (arrows).

Four-month old transformants were screened by PCR using gene-specific primers amplifying 373 bp of the *bar* gene coding region (figure S7A) and 837 bp of the *xylanase* gene and its *Hor5* promoter (figure S7B). Six of the 28 Chris and five of the 10 WED202-16-2 plants that survived bialaphos treatment after transformation contained the *xylanase* gene were identified. Six of these (5 in WED 202-16-2 and 1 in Chris) 11 transformants produced spontaneously doubled haploid seeds, and were analyzed further. For a proof that the transgenes were incorporated into the wheat chromosomes, the first intron of the *ubiquitin* gene inserted between the promoter and the *bar* gene was analyzed. Reverse transcription PCR was performed on cDNA synthesized from mRNA of the two transformants MT1-B4 and MT1-1 with specific primers covering the *bar* gene, the intron and the promoter. The primers amplified from the plasmid a fragment of 1212 bp, while the expected 198 bp fragment was produced from the cDNA of the transformants (figure S8). This demonstrated that the intron was removed in the transformants after transcription of the transgene and that the PCR products are not due to presence of *Agrobacterium*. To confirm that the 837 bp-fragment amplified by PCR originated from the xylanase transgene, the fragment was purified, inserted into a pUC 18 vector and cloned in *E. coli* DH5*α* cells. The fragment from the purified plasmid of a clone was sequenced. The sequence was identical to that of the *xylanase* gene used for construction of plasmid RS128/Xyl (data not shown). DNA of thirteen randomly chosen T_1_ MT1-B4 seedlings analyzed by PCR with the xylanase specific primers contained the 837 bp fragment (figure 4A). This result indicates that the primary transformant is a homozygous doubled haploid plant and that the transgene is stably inherited in the T_1_ generation. The stable inheritance was verified for T_2_ seedlings of MT1-B4, MT1-1, MT1-10 and MT1-11 in WED202-16-2 background, and MT1-6 and MT1-8 in Chris background.

Southern blot analyses were performed with 13 T_1_ progenies derived from 6 different T_0_ transformants (figure 4B). The results showed that DNA samples numbered 1, 2, 8, 9, 11, 12 and 13 in figure 4B had unique hybridization bands (high molecular weight, on the very top), which the wild type samples lacked (negative controls, lane nos. 14 and 15). Thus, it can be concluded that transformants MT1-1 (lanes 1 and 2), MT1-6 (lanes 8 and 9) and MT1-B4 (lanes 11, 12 and 13) contain the introduced xylanase gene. The results on the progeny of T_0_ primary transformant, MT1-B4 (lanes 11, 12, and 13) in combination with the results of PCR and sequence analysis confirmed the homozygous status of the introduced *xylanase* gene (no segregation) in this transformant. Southern analysis confirmed T-DNA integration(s) in haploid genomes of the 7 T_1_ seedlings representing 3 transformation events analyzed in this study. The Southern blot results verify the PCR and sequence results.

To determine the activity of xylanase in the doubled haploid transgenic grains a zymogram plate assay [46] was employed. The agar medium contained oat-spelt xylans that can be stained by Congo Red and are de-polymerized by the *Bacillus subtilis* 1,4-β-xylanase to stain-less products. As exemplified in figure 4C transgenic T_2_ wheat half grains containing the *xylanase* gene produced enzyme that diffused from the endosperm into the agar and caused an un-stainable circular area, while wild type grains lacked this capacity. The amount of 1,4-xylanase in grains of six T_2_ transgenic lines and their wild type cultivars was determined in extracts with the Megazyme assay using Brilliant BlueR linked azoxylan as substrate for extracts of flour grains. Conversion of the absorbance measured in the assay into µg of enzyme was obtained by calibration with pure 1,4-β-xylanase (cf. Materials & Methods). Both wheat cultivars contain endogenous xylanase. The four transgenic lines in cultivar WED202-16-2 contained twice the amount of xylanase than the host, and the two transgenic lines in Chris contained 1.7 times the enzyme amount than the host (table S11). This indicates a significant contribution of recombinant 1,4-β-xylanase in the transgenic grains.

### Prospect

Uninucleate microspores can be transformed by electroporation or co-cultivation with *A. tumefaciens* and regenerated into doubled haploid transgenic wheat plants. The method provides the possibility to introduce a single or several transgenes in homozygous form into an elite spring wheat cultivar in 8 months and in winter wheat cultivars in 16 months. In the present work a double-cassette vector (RS 128/Xyl) has been used, which similar to barley double cassette vectors can insert the selectable marker gene and the targeted gene in different genomic locations and thus permits the marker to be separated from the target gene by segregation of the heterozygous primary transformants [43], [65]. In the doubled haploid transgenic wheat plants this will require crosses with the host cultivar to remove the selection marker. There is room for further improvement of the method especially by modification of the selection marker design. Visual screening markers such as GFP may be used to aid the identification/selection of transgenic embryoids.

The primary objective of this study was to examine, if microspore transformation with subsequent androgenesis is possible in wheat. The transformants expressing 1,4-β-xylanase or *Trichoderma harzianum* endochitinase, generated to demonstrate the feasibility of the two microspore-based transformation techniques are of practical interest. The xylanase depolymerizes the major endosperm cell wall component of wheat grains, such as the arabinoxylan or pentosan chains of (1→4)- β-D-xylose molecules with α-L-arabinose side chains attached to the xylose by (1→2) and/or (1→3) linkages. Similarly, endochitinase degrade chitin in fungal cell walls providing resistance against root pathogenic fungi like *Rhizoctonia solani* and *Fusarium pseudograminearum*. Identifying transformants expressing large quantities of 1,4-β-xylanase in grains and endochitinase in roots will be the next steps to develop wheat lines with high nutritive values and showing resistance against root pathogenic fungi.

More recently an advancement of the ‘Agrolistic’ method standardized in maize was developed and tested in Triticale. The ‘agrolistic’, method combines the benefits of the *Agrobacterium* transformation system with that of the biolistic DNA delivery method. In the agrolistic method the virulence genes *vir*D1 and *vir*D2 from *A. tumefaciens* were placed under the control of a constitutive promoter and co-delivered to the morphogenic tissue with the target plasmid containing border sequences flanking the gene of interest [66]. Similarly, in a new transgene delivery method an *in vitro*-prepared nano-complex consisting of T-DNA, VirD2, and recombination protein A (RecA) was delivered to microspores with the help of a Tat2 cell-penetrating peptide [66]. Interestingly this method combines the benefits of both the *Agrobacterium*-based transformation system with the microspore based transformation procedure.

The agrolistic and *in vitro*-prepared nano-complex procedures are great advances in the field of plant transformation. The former approach was first reported in 1996, but has never gained much popularity since its inception, whereas the latter method was recently discovered and still requires further optimization. The methods presented in the present communication make use of the pre-established procedures, i.e., use of uninucleate androgenic microspores as explants and electroporation or co-cultivation with *Agrobacterium* as transfection procedures. Yet the presented procedures are quite innovative, and their novelty lies in the combination of transfection procedure adapted to obtain stable transformants with wheat microspores that can be obtained in large numbers from a limited number of the donor plants.

## Supporting Information

Figure S1 Wheat spikes at different developmental stages are indicative of microspore developmental stages: (a) 25% of spike exposed out of the flag leaf indicative of bi-nucleate microspores with a generative and a vegetative nucleus (too old); (b) spike with 3–5 cm slit in boot indicative of microspores with a single haploid nucleus (optimal).
(PDF)

Figure S2 Expression vectors used to demonstrate wheat transformations. (A) Single cassette vector pRB107 containing the *GUS* gene driven by the CAMV *35S* promoter. (B) Single cassette vector pRB113 containing the codon optimized endochitinase gene (from *Trichoderma harzianum*) driven by the CAMV *35S* promoter. (C) Single cassette vector pUbi.GFP containing the *GFP* gene driven by the maize *ubiquitin* promoter. (D) Double cassette vector RS 128/Xyl contains the *bar* gene driven by the *ubiquitin* promoter between one set of T-DNA left and right borders and the codon-optimized target gene for 1,4-β-xylanase driven by the hordein D gene promoter and supplied with the signal peptide between a second set of T-DNA borders.
(PDF)

Figure S3 Magnified view of microspores (section) undergoing three developmental pathways. ex =  exine wall, in =  intine layer, mt =  mitochondria, gl =  Golgi apparatus, p =  proplastid, rer =  rough endoplasmic reticulum, st =  starch.
(EPS)

Figure S4 A–C. Effect of different transduction and culture conditions on the number of green regenerants recovered from the microspore culture of 6–8 wheat spikes in regeneration/differentiation media. GEM =  germination of embryo of monocot [67], Mod. LS media  =  basal Linsmaier and Skoog medium [68] supplemented with 0.5 mg/L nicotinic acid, 0.5 mg/L pyridoxine hydrochloride, 0.5 mg/L kinetin, 0.2 mg/L phenylacetic acid, and 250 mg/L CuSO_4_.5H_2_O, and MMS5 + CuSO_4_.5H_2_O  =  Modified Murashige and Skoog (MS) Medium 5 [69] supplemented with 0.5 mg/L CuSO_4_.5H_2_O.
(PDF)

Figure S5 Molecular characterization of plants transformed with (A) pRB107 and (B) pRB113. For pRB107 two primer pairs 3′pHor-GUSNosF + Gus_PCR_R and GUS_F+5.Bar-GusR are uniquely capable of detecting transgene integration (top right). The former primer pair was used further for PCR based confirmation of transgene integration at T_0_, T_1_ and T_2_ generations (bottom left). Another primer pair GUS F and R was used for RT-PCR on the T_1_ and T_2_ cDNAs to confirm transgene expression (bottom right). Integration of transgene was also confirmed by sequencing of the PCR products (top left). In the line diagram, p107 represents the vector sequence, GUS F and R represent the primers used to amplify *GUS* gene from the wheat genomic DNA/plasmid DNA and T1_D6B, T0_50, T0_49, T0_D6 and T0_D7 represent the PCR products amplified from the genomic DNA of transformants and their progenies. A small part of the sequences was magnified to show integration of transgene in the wheat genome. For pRB113 a primer pair ThEndochit_F + pUbiGFPcheckbR is uniquely capable of detecting transgene integrations (top right). Thus, it was used further for PCR based confirmation of transgene integration at T_0_, T_1_ and T_2_ generations (bottom left). Another primer pair EndochitCheck F and R was used for RT-PCR on the T_1_ and T_2_ cDNAs to confirm transgene expression (bottom right). Integration of transgene was also confirmed by sequencing of the PCR products (top left). In the line diagram, pRB113 represents the vector sequence, T0_A3, T0_O1 and T1_O1A represent the PCR products amplified from the genomic DNA of transformants and their progenies. A small part of the sequences was magnified to show integration of transgene in the wheat genome. Endochitinase activity was also detected in transformants by fluorometric assay using methylumbelliferyl-chitotrioside (Sigma) as substrate (top extreme right). For primer details, see table S3; M = 100 bp ladder.
(PDF)

Figure S6 Chromosome complement of euhexaploid wheat cells derived from a pUbi.GFP transformed plant. (A) A root cell at mitotic metaphase showing 42 chromosomes. Chromosomes numbered 1, 13, 16 and 37 represent satellite chromosomes 1B1B and 6B6B. (B) A microspore mother cell at meiotic metaphase I showing 21 bivalents. The bivalents numbered 1 and 13 represent rod-bivalents and the rest represent ring-bivalents. The pictures were taken at 63× magnification.
(PDF)

Figure S7 PCR analysis of primary transformants for identification of the *bar* and *xylanase* genes. (A) Lanes: 1 = 1 kb ladder; 2 to 10 = 9 T_0_ transformants; 11 =  plasmid DNA. A 373 bp band was produced for the *bar* gene. (B) Lanes: 1 = H_2_O; 2 =  wild type wheat DNA; 3 = 1 kb DNA ladder; 4 =  plasmid DNA; 5 to 8 = DNA of T_0_ transformant ‘MT1 B4’ in WED 202-16-2 background at variable concentrations 200, 100, 50, and 20 ng/µl. A 837 bp band was amplified from the *xylanase* gene.
(PDF)

Figure S8 Reverse transcription PCR analysis for the *bar* gene to show removal of the 198 bp intron attached to its ubiquitin gene promoter for expression of the *bar* gene. Lanes: 1, 2 = cDNA of two wheat transformants; 3 = plasmid DNA; 4 = 100 bp ladder. RNA was derived from the developing T_1_ grains. A DNA fragment of 198 bp was amplified from the cDNA of the transformants, while a 1212 bp band was produced with plasmid DNA. The gel shift was due to removal of the intron.
(PDF)

Table S1 Composition of induction medium NPB-99 and regeneration medium 190-2.
(DOCX)

Table S2 Composition of MMS5 (pH5.8) regeneration media. The MMS5 medium was modified from original MS media for embryo differentiation [68].
(DOCX)

Table S3 List of primers used to confirm transgene integration and for RT-PCR.
(DOCX)

Table S4 Effects of genotype on the percentage of green regenerants obtained from embryo-like structures formed from the transfected androgenic microspores.
(DOCX)

Table S5 Effect on androgenesis of Chris microspores by concentration of *A. tumefaciens* in the medium for 24 hrs before filtration and addition of 200 mg·L^-1^ timentin.
(DOCX)

Table S6 Effect on androgenesis of Chris microspores by co-cultivation with 20% MT1 for varying duration (minutes) before filtration and addition of 200 mg·L^-1^ timentin.
(DOCX)

Table S7 Effect of timentin in the culture medium on Chris microspore androgenesis.
(DOCX)

Table S8 Effect of increasing concentrations of bialaphos used in the regeneration medium on the wild type Chris embryoids.
(DOCX)

Table S9 Staggered selection with bialaphos of wild type NPBCT embryoids for plant regeneration on 190-2 medium with different bialaphos concentrations.
(DOCX)

Table S10 Staggered selection with bialaphos of Chris and WED202-16-2 embryoids derived from transformed microspores for plant regeneration on 190-2 medium with different bialaphos concentrations.
(DOCX)

Table S11 Xylanase activity in 200 mg of flour from wild type and transgenic T_2_ wheat grains. Each assay was replicated thrice.
(DOCX)

## References

[1] Vasil V, Castillo AM, Fromm ME, Vasil IK (1992) Herbicide resistant fertile transgenic wheat plants obtained by microprojectile bombardment of regenerable embryogenic callus. Bio/Technology 10: 667–674.

[2] Vasil V, Srivastava V, Castillo AM, Fromm ME, Vasil IK (1993) Rapid production of transgenic wheat plants by direct bombardment of cultured immature embryos. Bio/Technology 11: 1553–1558.

[3] Cheng M, Fry JE, Pang S, Zhou H, Hironaka CM, et al (1997) Genetic transformation of wheat mediated by *Agrobacterium tumefaciens* . Plant Physiology 115: 971–980. 12223854 10.1104/pp.115.3.971 PMC158560

[4] Zhou H, Berg JD, Blank SE, Chay CA, Chen G, et al (2003) Field efficacy assessment of transgenic Roundup Ready wheat. Crop Science 43: 1072–1075.

[5] Pellegrineshi A, Noguera LM, Skovmand B, Brito RM, Velazquez L, et al (2002) Identification of highly transformable wheat genotypes for mass production of fertile transgenic plants. Genome 45: 421–430. 11962639 10.1139/g01-154

[6] Touraev A, Prosser M, Heberle-Bers E (2001) The microspore: a haploid multipurpose cell. Advances in Botanical Research 35: 53–109.

[7] Resch T, Touraev A (2011) Pollen transformation technologies. In: Stewart N, Touraev A, Citovsky V, Tzfira T, editors. Plant Transformation Technologies. Wiley-Blackwell. pp. 83–91.

[8] Kumlehn J, Serazetdinova L, Hensel G, Becker D, Loerz H (2006) Genetic transformation of barley (*Hordeum vulgare* L.) via infection of androgenetic pollen cultures with *Agrobacterium tumefaciens* . Plant Biotechnology Journal 4: 251–261. 17177801 10.1111/j.1467-7652.2005.00178.x

[9] Kumlehn J (2009) Embryogenic pollen culture: A promising target for genetic transformation. In: Touraev A, Forster BP, Jain SM, editors. Advances in Haploid Production in Higher Plants. Springer. pp. 295–305.

[10] Haliloğlu K, Baenziger PS (2002) Optimization of wheat (*Triticum aestivum* L.) anther culture-derived embryos transformation by microprojectile bombardment. Atatürk Üniversitesi Ziraat Fakültesi Dergisi 33: 413–416.

[11] Barcelo P, Lazzeri P (1998) Direct gene transfer: Chemical, electrical and physical methods. In: Lindsey K, editors. Transgenic Plant Research. Harwood Academic Press, The Netherlands. pp 35–55.

[12] Brisibe EA, Gajdosova A, Olesen A, Anderson SB (2000) Cytodifferentiation and transformation of embryogenic callus lines derived from anther culture of wheat. Journal of Experimental Botany 51: 187–196. 10938825 10.1093/jexbot/51.343.187

[13] Folling L, Olesen A (2001) Transformation of wheat (*Triticum aestivum* L.) microspore-derived callus and microspores by particle bombardment. Plant Cell Reports 20: 629–636.

[14] Massiah A, Rong H, Brown S, Laurie S (2001) Accelerated production and identification of, homozygous transgenic wheat lines by anther culture. Molecular Breeding 7: 163–173.

[15] Gharanjik S, Moieni A, Mousavi A, Alizadeh H (2008) Optimization of transient expression of *uidA* gene in androgenic embryos of wheat (*Triticum aestivum* L. cv Falat) via particle bombardment. Iranian Journal of Biotechnology 6: 207–213.

[16] Chauhan H, Khurana P (2011) Use of doubled haploid technology for development of stable drought tolerant bread wheat (*Triticum aestivum* L.) transgenics. Plant Biotechnology Journal 9: 408–417. 20723133 10.1111/j.1467-7652.2010.00561.x

[17] Navarro-Alvarez W, Baenziger PS, Eskridge KM, Hugo M, Gustafson VD (1994) Addition of colchicine to wheat anther culture media to increase doubled haploid production. Plant Breeding 112: 192–198.

[18] Stober A, Hess D (1997) Spike pretreatments, anther culture conditions and anther culture response of seventeen German varieties of spring wheat (*Triticum aestivum* L.). Plant Breeding 116: 443–447.

[19] Barnabás B (2003) Protocol for producing doubled haploid plants from anther culture of wheat (*Triticum aestivum* L.). In: Maluszynski M, Kasha KJ, Forster BP, Szarejko I, editors. Doubled haploid production in crop plants. A manual. Dordrecht, Boston, London, Kluwer Academic Publishers. pp 65–70.

[20] Joersbo M, Jorgensen RB, Olesen P (1990) Transient electropermeabilization of barley (*Hordeum vulgare* L.) microspores to propidium iodide. Plant Cell, Tissue and Organ Culture 23: 125–129.

[21] Fennell A, Hauptmann R (1992) Electroporation and PEG delivery of DNA into maize microspores. Plant Cell Reports 11: 567–570. 24213288 10.1007/BF00233094

[22] Jardinaud M-F, Souvre A, Beckert M, Alibert G (1995) Optimisation of DNA transfer and transient β-glucuronidase expression in electroporated maize (*Zea mays* L.) microspores. Plant Cell Reports 15: 55–58. 24185654 10.1007/BF01690253

[23] Obert B, Ponya Z, Pretova A, Barnabas B (2004) Optimization of electroporation conditions for maize microspores. Maydica 49: 15–19.

[24] Barclay IR (1975) High frequency production of haploids in wheat (*Triticum aestivum*) by chromosome elimination. Nature 256: 410–411.

[25] Laurie DA, Bennett MD (1988) The production of haploid wheat plants from wheat x maize crosses. Theoretical and Applied Genetics 76: 393–397. 24232203 10.1007/BF00265339

[26] Laurie DA, Bennett MD (1988) Cytological evidence for fertilization in hexaploid wheat x Sorghum crosses. Plant Breeding 100: 73–82.

[27] Ouyang TW, Hu H, Chuang CC, Tseng CC (1973) Induction of pollen plants from anthers of *Triticum aestivum* L. cultured *in vitro* . Scientia Sinica 16: 79–95.

[28] Touraev A, Indrianto A, Wratschko I, Vicente O, Heberle-Bors E (1996) Efficient microspore embryogenesis in wheat (*Triticum aestivum* L.) induced by starvation at high temperatures. Sexual Plant Reproduction 9: 209–215.

[29] Han H (1997) *In vitro* induced haploids in wheat. In: Jain SM, Sopory SK, Veilleux RE, editors. *In vitro* Haploid Production in Higher Plants. Kluwer Academic Publishers, Boston, Dordrecht. pp. 73–97.

[30] Zhou H, Konzak CF (1997) Influence of genetic and environmental factors on anther culture response of wheat. Journal of Applied Genetics 38: 393–406.

[31] Simonson RL, Baenziger PS, Gusafson D (1997) Wheat anther culture as affected by various cultural changes and supplements. Journal of Applied Genetics 38: 381–392.

[32] Hu T, Kasha KJ (1997) Improvement of isolated microspore culture in wheat (*Triticum aestivum* L.) through ovary co-culture. Plant Cell Reports 16: 520–525. 10.1007/BF01142316 30727571

[33] Hu T, Kasha KJ (1999) A cytological study of pretreatments used to improve isolated microspore cultures of wheat (*Triticum aestivum* L.) cv Chris. Genome 42: 432–441.

[34] Mentewab A, Letellier V, Marque C, Sarrafi A (1999) Use of anthocyanin biosynthesis stimulatory genes as markers for the genetic transformation of haploid embryos and isolated microspores in wheat. Cereal Research Communications 27: 17–24.

[35] Kumar M (2004) Genetic modification of wheat (*Triticum aestivum* L.) straw for potential use as a biofuel. M.Sc. thesis, University of Idaho, Moscow, ID.

[36] Liu W, Zheng M, Polle E, Konzak CF (2002) Highly efficient doubled-haploid production in wheat (*Triticum aestivum* L.) via induced microspore embryogenesis. Crop Science 42: 686–692.

[37] Liu W, Zheng M, Konzak CF (2002) Improving green plant production via isolated microspore culture in bread wheat (*Triticum aestivum* L.). Plant Cell Reports 20: 821–824.

[38] Zheng MY, Liu W, Weng Y, Polle E, Konzak CF (2003) Production of doubled haploids in wheat (*Triticum aestivum* L.) through microspore embryogenesis triggered by inducer chemicals. In: Maluszynski M, Kasha KJ, Froster BP, Szarejko I, editors. Doubled haploid production in crop plants, Kluwer Academic Publishers. pp 83–94.

[39] Zheng MY, Weng Y, Liu W, Konzak CF (2001) The effect of ovary-conditioned medium on microspore embryogenesis in common wheat (*Triticum aestivum* L.). Plant Cell Reports 20: 802–807.

[40] Kohl EA (2003) Development of transgenic barley expressing (1,4)- β-xylanase. M.Sc. Thesis, Dept. Crop and Soil Sciences. Washington State University, Pullman WA pp. 36–58.

[41] Horvath H, Huang J, Wong O, Kohl E, Okita T, et al (2000) The production of recombinant proteins in transgenic barley grains. Proceedings of the National Academy of Sciences of the United States of America 97: 1914–1919. 10677555 10.1073/pnas.030527497 PMC26536

[42] Sørensen MB, Müller M, Skerrit J, Simpson D (1996) Hordein promoter methylation and transcriptional activity in wild type and mutant barley endosperm. Molecular and General Genetics 250: 750–760. 8628236 10.1007/BF02172987

[43] Horvath H, Huang J, Wong OT, von Wettstein D (2002) Experiences with genetic transformation of barley and characteristics of transgenic plants. In: Slafer GA, Molina-Cano JL, Savin R, Araus JL, Romagosa I, editors. Barley Science. The Harworth Press, New York. pp. 143–176.

[44] Lee H, Rustgi S, Kumar N, Burke I, Yenish JP, et al (2011) Single nucleotide mutation in the barley *acetohydroxy acid synthase* (*AHAS*) gene confers resistance to imidazolinone herbicides. Proceedings of the National Academy of Sciences of the United States of America 108: 8909–8913. 21551103 10.1073/pnas.1105612108 PMC3102390

[45] Kleinhofs A, Kilian A, Saghai Maroof MA, Biyashev RM, Hayes P, et al (1993) A molecular, isozyme and morphological map of the barley (*Hordeum vulgare*) genome. Theoretical and Applied Genetics 86: 705–712. 24193780 10.1007/BF00222660

[46] Sa-Pereira P, Costa-Ferreira M, Aires-Barros MR (2002) Enzymatic properties of a neutral endo-1,3(4) β-xylanase Xyl II from *Bacillus subtilis* . Journal of Biotechnology 94: 265–275. 11861085 10.1016/s0168-1656(01)00436-9

[47] McCabe DE, Swain WF, Martinelli BJ, Christou P (1988) Stable transformation of soybean (*Glycine max*) by particle acceleration. Bio/Technology 6: 923–926.

[48] Sharma AK, Sharma A (1980) Chromosome techniques: theory and practice. Butterworths, London

[49] Maraschin SD, Vennik M, Lamers GEM, Spaink HP, Wang M (2005) Time-lapse tracking of barley androgenesis reveals position-determined cell death within pro-embryos. Planta 220: 531–540. 15449059 10.1007/s00425-004-1371-x

[50] Maraschin SF, de Priester W, Spaink HP, Wang M (2005) Androgenic switch: an example of plant embryogenesis from the male gametophyte perspective. Journal of Experimental Botany 56: 1711–1726. 15928015 10.1093/jxb/eri190

[51] Touraev A, Vicente O, Heberle-Bors E (1997) Initiation of microspore embryogenesis by stress. Trends in Plant Science 2: 297–302.

[52] Indrianto A, Barinova I, Touraev A, Heberle-Bors E (2001) Tracking individual wheat microspores *in vitro*: Identification of embryogenic microspores and body axis formation in the embryo. Planta 212: 163–174. 11216836 10.1007/s004250000375

[53] Jacquard C, Mazeyrat-Gourbeyer F, Devaux P, Boutilier K, Baillieul F, et al (2009) Microspore embryogenesis in barley: anther pre-treatment stimulates plant defense gene expression. Planta 229: 393–402. 18974997 10.1007/s00425-008-0838-6

[54] Maraschin SF, Lamers GEM, de Pater BS, Spaink HP, Wang M (2003) 14-3-3 isoforms and pattern formation during barley microspore embryogenesis. Journal of Experimental Botany 51: 1033–1043. 10.1093/jxb/erg098 12598573

[55] Torp AM, Andersen SB (2009) Albinism in microspore culture. In: Touraev A, Forster BP, Jain SM, editors. Advances in Haploid Production in Higher Plants. Springer. pp. 155–160.

[56] Pedersen C, Zimny J, Becker D, Janhne-Gartner A, Lorz H (1997) Localization of introduced genes on the chrmomosomes of transgenic barley, wheat and triticale by fluorescence in situ hybridization. Theoretical and Applied Genetics 94: 749–757.

[57] Svitashev S, Ananiev E, Pawlowski WP, Somers DA (2000) Association of transgene integration sites with chromosome rearrangements in hexaploid oat. Theoretical and Applied Genetics 100: 872–880.

[58] Feldmann KA (1991) T-DNA insertion mutagenesis in Arabidopsis - mutational spectrum. Plant Journal 1: 71–82.

[59] Lindsey K, Wei WB, Clarke MC, McArdle HF, Rooke LM, et al (1993) Tagging genomic sequences that direct transgene expression by activation of a promoter trap in plants. Transgenic Research 2: 33–47. 8513337 10.1007/BF01977679

[60] Dong JJ, Kharb P, Teng WM, Hall TC (2001) Characterization of rice transformed via an Agrobacterium-mediated inflorescence approach. Molecular Breeding 7: 187–194.

[61] Irish VF (1991) Cell lineage in plant development. Current Opinion in Genetics & Development 1: 169–173. 1822266 10.1016/s0959-437x(05)80065-6

[62] Li B, Xie C, Qiu H (2009) Production of selectable marker-free transgenic tobacco plants using a non-selection approach: chimerism or escape, transgene inheritance, and efficiency. Plant Cell Reports 28: 373–386. 19018535 10.1007/s00299-008-0640-8

[63] Bahieldin A, Eissa HF, Mahfouz HT, Dyer WE, Madkour MA, et al (2005) Evidence for non-proteinaceous inhibitor(s) of β-glucuronidase in wheat (*Triticum aestivum* L.) leaf and root tissues. Plant Cell, Tissue and Organ Culture 82: 11–17.

[64] Stahl R, Horvath H, Van Fleet J, Voetz M, von Wettstein D, et al (2002) T-DNA integration into the barley genome from single and double cassette vectors. Proceedings of the National Academy of Sciences of the United States of America 99: 2146–2151. 11854511 10.1073/pnas.032645299 PMC122333

[65] Hansen G, Shillito RD, Chilton MD (1997) T-strand integration in maize protoplasts after codelivery of a T-DNA substrate and virulence genes. Proceedings of the National Academy of Sciences of the United States of America 94: 11726–11730. 9326678 10.1073/pnas.94.21.11726 PMC23615

[66] Ziemienowicz A, Shim Y-S, Matsuoka A, Eudes F, Kovalchuk I (2012) A novel method of transgene delivery into Triticale plants using the *Agrobacterium* transferred DNA-derived nano-complex. Plant Physiology 158: 1503–1513. 22291201 10.1104/pp.111.192856 PMC3320166

[67] Eudes F, Acharya S, Laroche A, Selinger LB, Cheng K-J (2003) A novel method to induce direct somatic embryogenesis, secondary embryogenesis and regeneration of fertile green cereal plants. Plant Cell, Tissue and Organ Culture 73: 147–157.

[68] Linsmaier EM, Skoog F (1965) Organic growth factor requirements of tobacco tissue culture. Physiologia Plantarum 18: 100–127.

[69] Kasha KJ, Simion E, Miner M, Letarte J, Hu TC (2003) Haploid wheat isolated microspore culture protocol. In: Maluszynski, Kasha KJ, Forster BP, Szarejko I, editors. Doubled haploid production in crop plants. Kluwer Academic Publishers, The Netherlands. pp. 77–82.

