# Supplementary figures and images for: Generation of Doubled Haploid Transgenic Wheat Lines by Microspore Transformation
Source: PLoS One. 2013 Nov 18;8(11):e80155. doi: 10.1371/journal.pone.0080155 (PMC3832437; doi:10.1371/journal.pone.0080155)

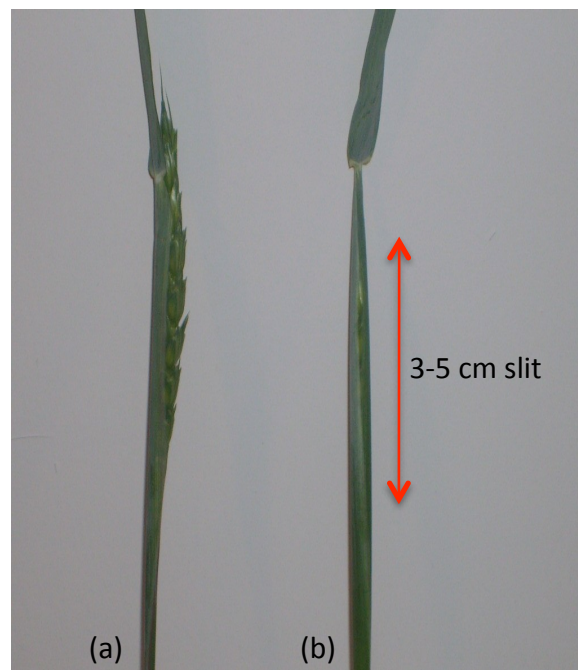

Supplement: Figure S1 — Wheat spikes at different developmental stages are indicative of microspore developmental stages: (a) 25% of spike exposed out of the flag leaf indicative of bi-nucleate microspores with a generative and a vegetative nucleus (too old); (b) spike with 3–5 cm slit in boot indicative of microspores with a single haploid nucleus (optimal). (PDF) [file pone.0080155.s001.pdf]

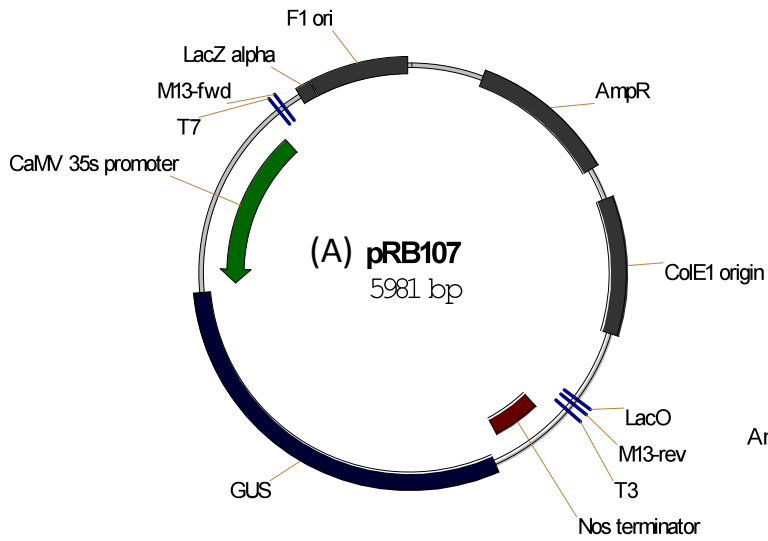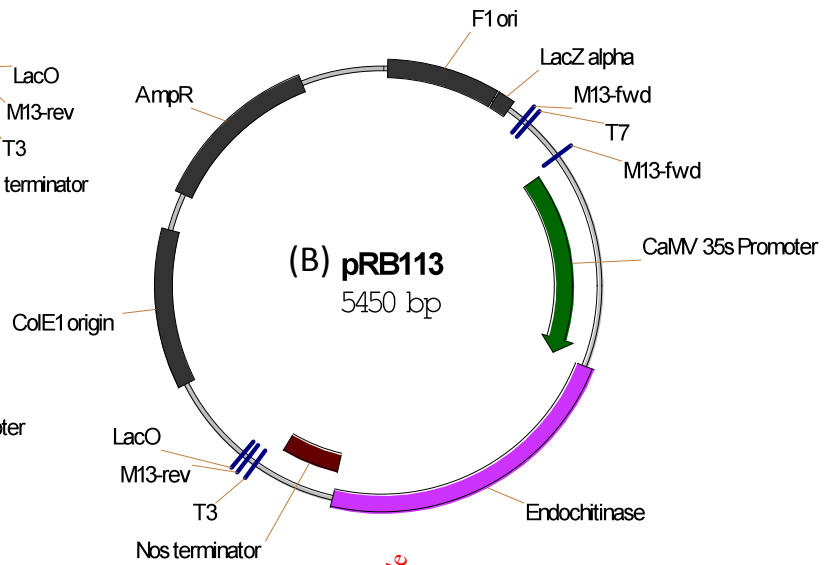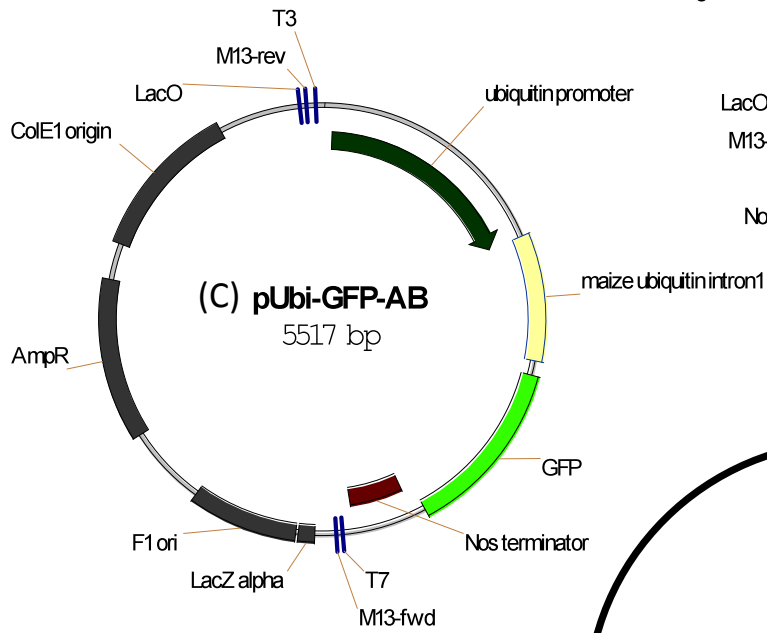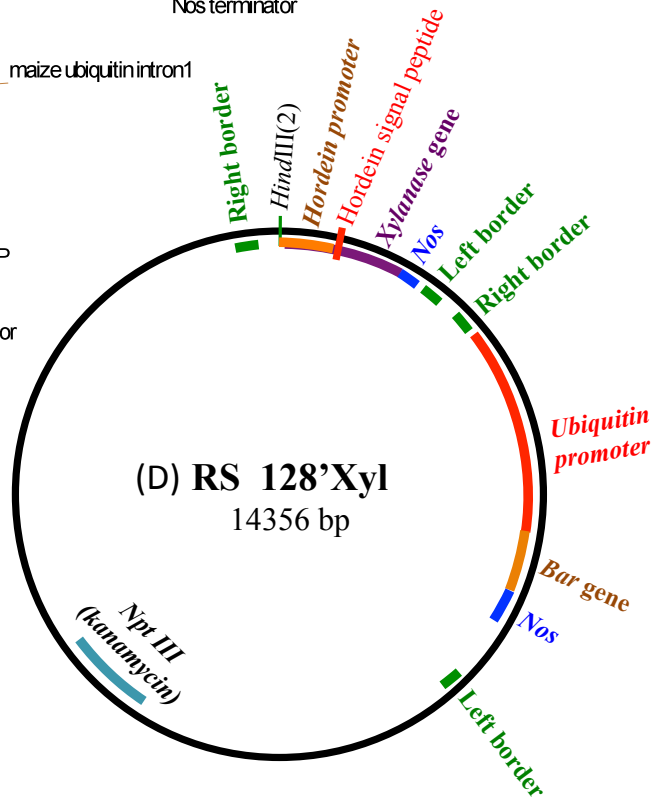

Supplement: Figure S2 — Expression vectors used to demonstrate wheat transformations. (A) Single cassette vector pRB107 containing the GUS gene driven by the CAMV 35S promoter. (B) Single cassette vector pRB113 containing the codon optimized endochitinase gene (from Trichoderma harzianum) driven by the CAMV 35S promoter. (C) Single cassette vector pUbi.GFP containing the GFP gene driven by the maize ubiquitin promoter. (D) Double cassette vector RS 128/Xyl contains the bar gene driven by the ubiquitin promoter between one set of T-DNA left and right borders and the codon-optimized target gene for 1,4-β-xylanase driven by the hordein D gene promoter and supplied with the signal peptide between a second set of T-DNA borders. (PDF) [file pone.0080155.s002.pdf]

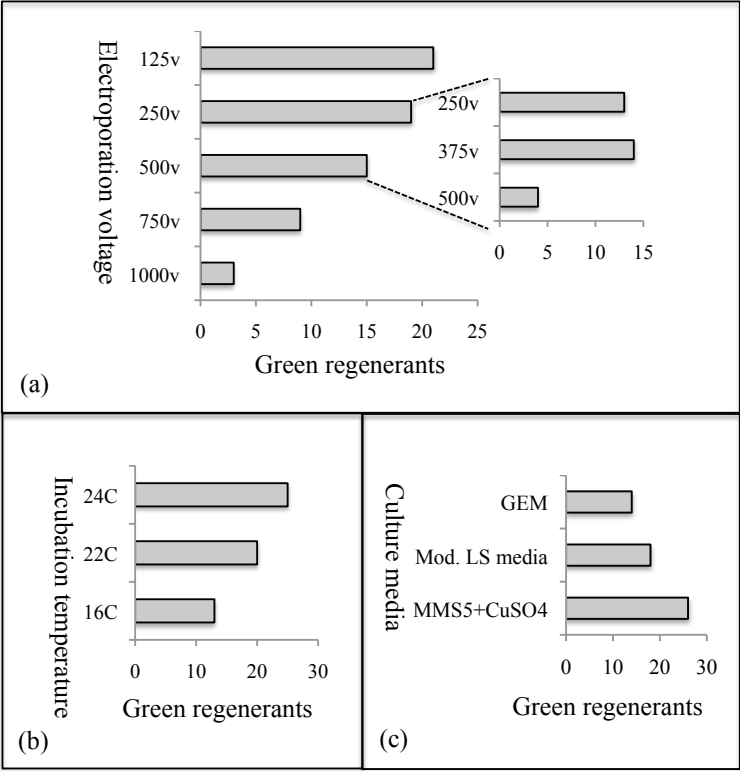

Supplement: Figure S4 — A–C. Effect of different transduction and culture conditions on the number of green regenerants recovered from the microspore culture of 6–8 wheat spikes in regeneration/differentiation media. GEM = germination of embryo of monocot [67], Mod. LS media = basal Linsmaier and Skoog medium [68] supplemented with 0.5 mg/L nicotinic acid, 0.5 mg/L pyridoxine hydrochloride, 0.5 mg/L kinetin, 0.2 mg/L phenylacetic acid, and 250 mg/L CuSO4.5H2O, and MMS5 + CuSO4.5H2O = Modified Murashige and Skoog (MS) Medium 5 [69] supplemented with 0.5 mg/L CuSO4.5H2O. (PDF) [file pone.0080155.s004.pdf]

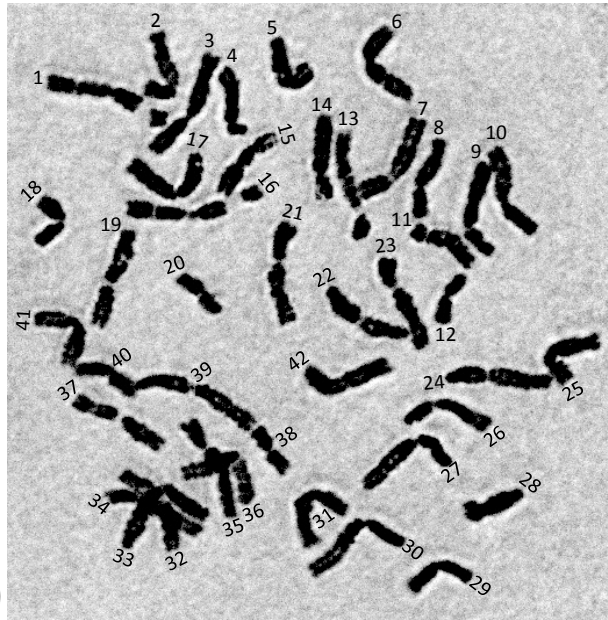

(a)

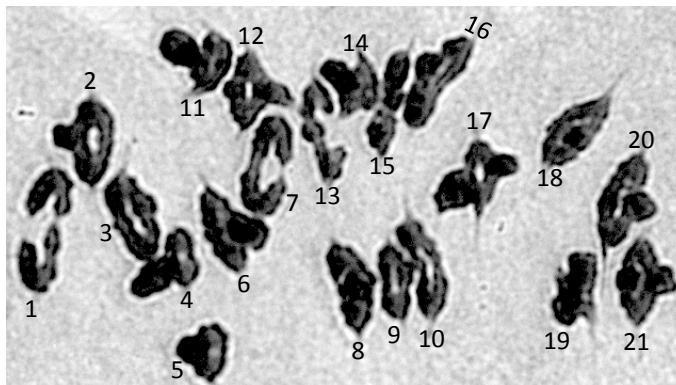

(b)

Supplement: Figure S6 — Chromosome complement of euhexaploid wheat cells derived from a pUbi.GFP transformed plant. (A) A root cell at mitotic metaphase showing 42 chromosomes. Chromosomes numbered 1, 13, 16 and 37 represent satellite chromosomes 1B1B and 6B6B. (B) A microspore mother cell at meiotic metaphase I showing 21 bivalents. The bivalents numbered 1 and 13 represent rod-bivalents and the rest represent ring-bivalents. The pictures were taken at 63× magnification. (PDF) [file pone.0080155.s006.pdf]

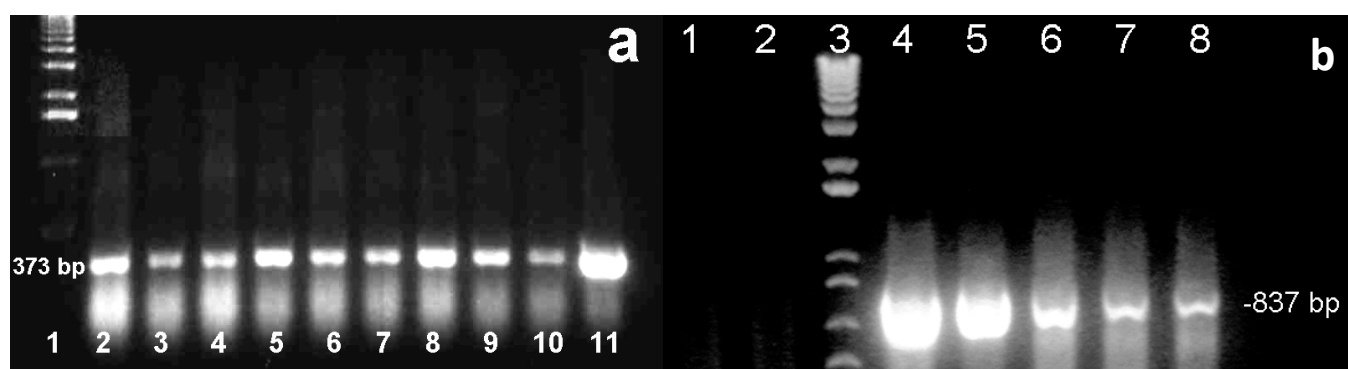

Supplement: Figure S7 — PCR analysis of primary transformants for identification of the bar and xylanase genes. (A) Lanes: 1 = 1 kb ladder; 2 to 10 = 9 T0 transformants; 11 = plasmid DNA. A 373 bp band was produced for the bar gene. (B) Lanes: 1 = H2O; 2 = wild type wheat DNA; 3 = 1 kb DNA ladder; 4 = plasmid DNA; 5 to 8 = DNA of T0 transformant ‘MT1 B4’ in WED 202-16-2 background at variable concentrations 200, 100, 50, and 20 ng/µl. A 837 bp band was amplified from the xylanase gene. (PDF) [file pone.0080155.s007.pdf]

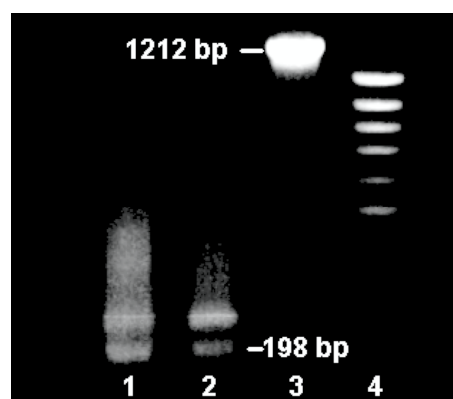

Supplement: Figure S8 — Reverse transcription PCR analysis for the bar gene to show removal of the 198 bp intron attached to its ubiquitin gene promoter for expression of the bar gene. Lanes: 1, 2 = cDNA of two wheat transformants; 3 = plasmid DNA; 4 = 100 bp ladder. RNA was derived from the developing T1 grains. A DNA fragment of 198 bp was amplified from the cDNA of the transformants, while a 1212 bp band was produced with plasmid DNA. The gel shift was due to removal of the intron. (PDF) [file pone.0080155.s008.pdf]
