# Supplementary material for: Generation of Doubled Haploid Transgenic Wheat Lines by Microspore Transformation
Source: PLoS One. 2013 Nov 18;8(11):e80155. doi: 10.1371/journal.pone.0080155 (PMC3832437; doi:10.1371/journal.pone.0080155)
Supplement: Figure S5 — Molecular characterization of plants transformed with (A) pRB107 and (B) pRB113. For pRB107 two primer pairs 3′pHor-GUSNosF + Gus_PCR_R and GUS_F+5.Bar-GusR are uniquely capable of detecting transgene integration (top right). The former primer pair was used further for PCR based confirmation of transgene integration at T0, T1 and T2 generations (bottom left). Another primer pair GUS F and R was used for RT-PCR on the T1 and T2 cDNAs to confirm transgene expression (bottom right). Integration of transgene was also confirmed by sequencing of the PCR products (top left). In the line diagram, p107 represents the vector sequence, GUS F and R represent the primers used to amplify GUS gene from the wheat genomic DNA/plasmid DNA and T1_D6B, T0_50, T0_49, T0_D6 and T0_D7 represent the PCR products amplified from the genomic DNA of transformants and their progenies. A small part of the sequences was magnified to show integration of transgene in the wheat genome. For pRB113 a primer pair ThEndochit_F + pUbiGFPcheckbR is uniquely capable of detecting transgene integrations (top right). Thus, it was used further for PCR based confirmation of transgene integration at T0, T1 and T2 generations (bottom left). Another primer pair EndochitCheck F and R was used for RT-PCR on the T1 and T2 cDNAs to confirm transgene expression (bottom right). Integration of transgene was also confirmed by sequencing of the PCR products (top left). In the line diagram, pRB113 represents the vector sequence, T0_A3, T0_O1 and T1_O1A represent the PCR products amplified from the genomic DNA of transformants and their progenies. A small part of the sequences was magnified to show integration of transgene in the wheat genome. Endochitinase activity was also detected in transformants by fluorometric assay using methylumbelliferyl-chitotrioside (Sigma) as substrate (top extreme right). For primer details, see table S3; M = 100 bp ladder. (PDF) [file pone.0080155.s005.pdf]

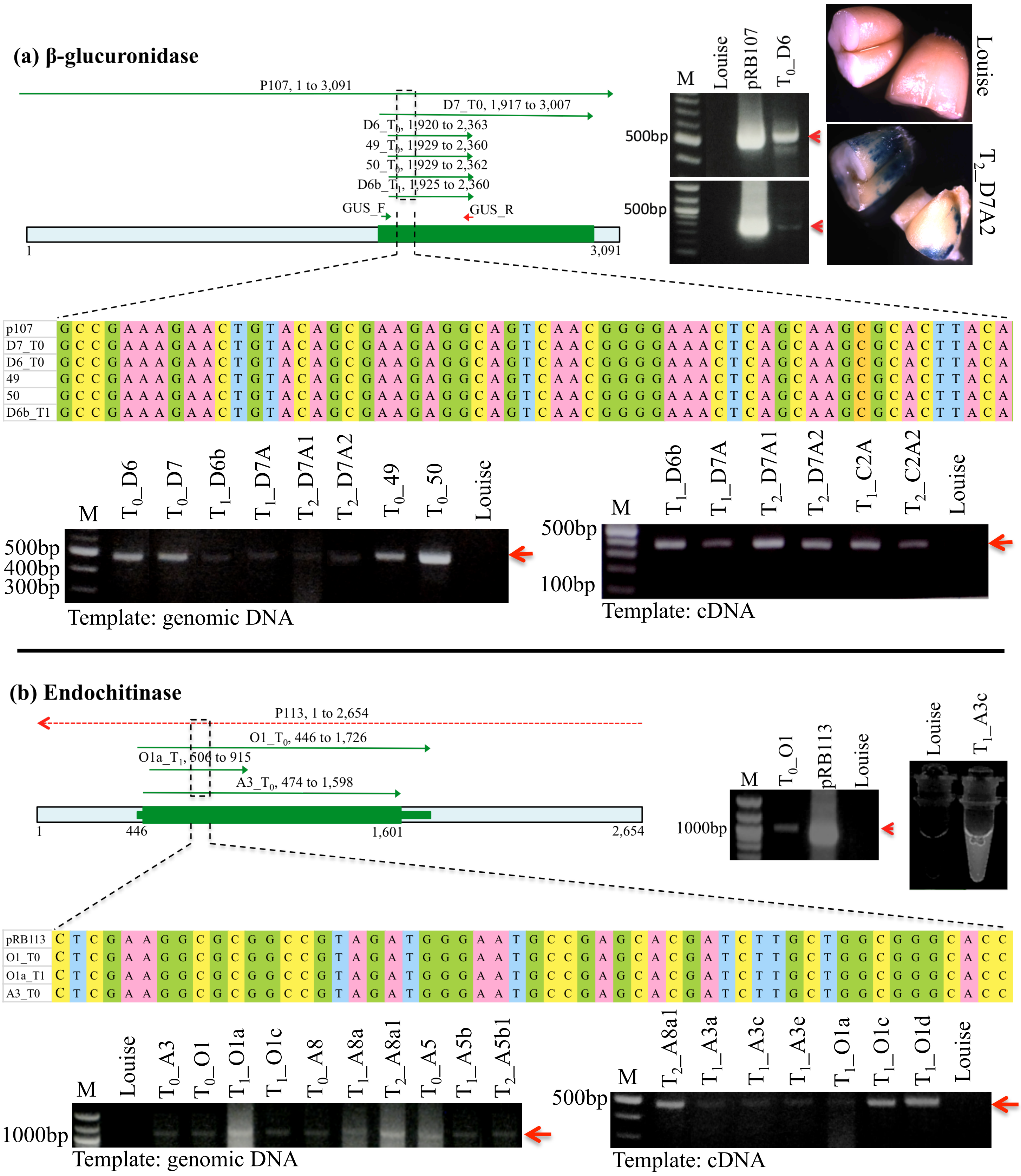

(a) β-glucuronidase
P107, 1 to 3,091
D7_T0, 1,917 to 3,007
D6_T0, 1,920 to 2,363
49_T0, 1,929 to 2,360
50_T0, 1,929 to 2,362
D6b_T1, 1,925 to 2,360
GUS_F
GUS_R
1
3,091
M
Louise
pRB107
T0_D6
500bp
500bp
Louise
T2_D7A2
p107 GCCGAAAGAACTGTACAGCGAAGAGGCAGTCAACGGGGAAACTCAGCAAGCGCACTTACA
D7_T0 GCCGAAAGAACTGTACAGCGAAGAGGCAGTCAACGGGGAAACTCAGCAAGCGCACTTACA
D6_T0 GCCGAAAGAACTGTACAGCGAAGAGGCAGTCAACGGGGAAACTCAGCAAGCGCACTTACA
49 GCCGAAAGAACTGTACAGCGAAGAGGCAGTCAACGGGGAAACTCAGCAAGCGCACTTACA
50 GCCGAAAGAACTGTACAGCGAAGAGGCAGTCAACGGGGAAACTCAGCAAGCGCACTTACA
D6b_T1 GCCGAAAGAACTGTACAGCGAAGAGGCAGTCAACGGGGAAACTCAGCAAGCGCACTTACA
M
T0_D6
T0_D7
T1_D6b
T1_D7A
T2_D7A1
T2_D7A2
T0_49
T0_50
Louise
500bp
400bp
300bp
Template: genomic DNA
M
T1_D6b
T1_D7A
T2_D7A1
T2_D7A2
T1_C2A
T2_C2A2
Louise
500bp
100bp
Template: cDNA
(b) Endochitinase
P113, 1 to 2,654
O1_T0, 446 to 1,726
O1a_T1, 506 to 915
A3_T0, 474 to 1,598
1
446
1,601
2,654
M
T0_O1
pRB113
Louise
1000bp
Louise
T1_A3c
pRB113 CTCGAAGGCGCGGCCGTAGATGGGAATGCCGAGCACGATCTTGCTGGCGGGCACC
O1_T0 CTCGAAGGCGCGGCCGTAGATGGGAATGCCGAGCACGATCTTGCTGGCGGGCACC
O1a_T1 CTCGAAGGCGCGGCCGTAGATGGGAATGCCGAGCACGATCTTGCTGGCGGGCACC
A3_T0 CTCGAAGGCGCGGCCGTAGATGGGAATGCCGAGCACGATCTTGCTGGCGGGCACC
M
Louise
T0_A3
T0_O1
T1_O1a
T1_O1c
T0_A8
T1_A8a
T2_A8a1
T0_A5
T1_A5b
T2_A5b1
1000bp
Template: genomic DNA
M
T2_A8a1
T1_A3a
T1_A3c
T1_A3e
T1_O1a
T1_O1c
T1_O1d
Louise
500bp
Template: cDNA
