# Supplementary material for: Generation of Doubled Haploid Transgenic Wheat Lines by Microspore Transformation
Source: PLoS One. 2013 Nov 18;8(11):e80155. doi: 10.1371/journal.pone.0080155 (PMC3832437; doi:10.1371/journal.pone.0080155)
Supplement: Table S1 — Composition of induction medium NPB-99 and regeneration medium 190-2. (DOCX) [file pone.0080155.s009.docx]

**Table S1.** Composition of induction medium NPB-99* and regeneration medium 190-2**.

|  | |  |  |
| --- | --- | --- | --- |
| **Component** | | **NPB-99**  **(mg/L)** | **190-2**  **(mg/L)** |
| Macro | (NH_4_)_2_SO_4_ | 232 | 200 |
|  | KNO_3_ | 1,415 | 1,000 |
|  | CaCl_2_⋅2H_2_O | 83 | - |
|  | Ca(NO_3_)_2_⋅4H_2_O | - | 100 |
|  | KH_2_PO_4_ | 200 | 300 |
|  | Mg(SO_4_)⋅7H_2_O | 93 | 200 |
|  | KCl | - | 40 |
| Micro | H_3_BO_3_ | 5 | 3 |
|  | CoCl_2_⋅6H_2_O | 0.0125 | - |
|  | CuSO_4_⋅5H_2_O | 0.0125 | - |
|  | KI | 0.40 | 0.50 |
|  | MnSO_4_⋅4H_2_O | 5 | 8 |
|  | Na_2_MoO_4_⋅2H_2_O | 0.0125 | - |
|  | ZnSO_4_⋅7H_2_O | 5 | 3 |
| Iron | Na_2_EDTA | 37.3 | 37.3 |
|  | FeSO_4_⋅7H_2_O | 27.8 | 27.8 |
| Amino Acids | Glycine | - | 2 |
| Vitamins | Pyridoxine HCl | 0.5 | 0.5 |
|  | Nicotinic Acid | 0.5 | 0.5 |
|  | Thiamine HCl | 5 | - |
|  | Glutamine | 500 | - |
| Sugars | myo-Inositol | 50 | 100 |
|  | Sucrose | - | 30,000 |
|  | Maltose | 90,000 | - |
| Hormone | Kinetin | 0.2 | - |
|  | 2,4-D | 0.2 | - |
|  | PAA | 1 | - |
| PH |  | 7.0 | 6.5 |
| Gelling | Phytagel | - | 4,000 |

Medium 190-2 is autoclaved at 240°F for 30 min.

*NPB-99 = Northwest Plant Breeding-99 [Zheng MY, Liu W, Weng Y, Polle E, Konzak CF (2003) Production of doubled haploids in wheat (*Triticum aestivum* L.) through microspore embryogenesis triggered by inducer chemicals. In: Maluszynski M, Kasha KJ, Froster BP, Szarejko I (eds.), Doubled haploid production in crop plants, Kluwer Academic Publishers, pp 83-94.]

**Wang XZ, Hu H (1984) The effect of potato II medium for triticale anther culture. Plant Science Letters 36: 237-239.
