# Supplementary material for: Generation of Doubled Haploid Transgenic Wheat Lines by Microspore Transformation
Source: PLoS One. 2013 Nov 18;8(11):e80155. doi: 10.1371/journal.pone.0080155 (PMC3832437; doi:10.1371/journal.pone.0080155)
Supplement: Table S2 — Composition of MMS5 (pH5.8) regeneration media. The MMS5 medium was modified from original MS media for embryo differentiation [68]. (DOCX) [file pone.0080155.s010.docx]

**Table S2.** Composition of Modified Murashige and Skoog 5 (MMS5*) regeneration/rooting media (pH5.8).

|  |  |
| --- | --- |
| **Component** | **mg/L** |
| Macro salts  1. KNO_3_  2. NH_4_NO_3_  3. KH_2_PO_4_  4. MgSO_4_.7H_2_O  5. CaCl_2_.2H_2_O | 1,400  300  170  370  440 |
| Iron source  FeSO_4_.7H_2_O  Na_2_ EDTA | 27.8  37.5 |
| Micro salts  MnSO_4_.H_2_O  H_3_BO_3_  ZnSO_4_.7H_2_O  CoCl.6H_2_O  CuSO_4_.5H_2_O  NaMoO_4_.2H_2_O  KI | 22.3  6.2  8.6  0.025  2.5  0.25  0.83 |
| Other components  Glutamine  Myo-inositol  Thiamine-HCL  Nicotinic acid  Pyroxidine HCl  Maltose monohydrate  PAA  Kinetin  Phytagel  GA_3_  Amino acids, U2.5 | 975  300  0.4  0.5  0.5  30,000  0.2  0.5  3,000  0.5  355 |

*Modified from the original MS media [cf. Kasha KJ, Simion E, Miner M, Letarte J, Hu TC (2003) Haploid wheat isolated microspore culture protocol. In: Maluszynski M, Kasha KJ, Froster BP, Szarejko I (eds.), Doubled haploid production in crop plants, Kluwer Academic Publishers, pp 77-81.]
