# Supplementary material for: Generation of Doubled Haploid Transgenic Wheat Lines by Microspore Transformation
Source: PLoS One. 2013 Nov 18;8(11):e80155. doi: 10.1371/journal.pone.0080155 (PMC3832437; doi:10.1371/journal.pone.0080155)
Supplement: Table S3 — List of primers used to confirm transgene integration and for RT-PCR. (DOCX) [file pone.0080155.s011.docx]

**Table S3.** List of primers used to confirm transgene integration and for RT-PCR.

|  |  |  |  |  |
| --- | --- | --- | --- | --- |
| **Name** | **Sequence** | **Am (C)*** | **Template** | **Product size (bp)** |
| pRB107 (GUS) |  |  |  |  |
| GUS_F | TTACGCTGAAGAGATGCTCGACTG | 61.1 | cDNA | 439 |
| GUS_R | TTCCAGTACCTTCTCTGCCGTTTC |  |  |  |
| 3’pHor-GusNosF | GTCCACCGAGATGTTACGTCCTGTAGAAACCC | 56.7 | gDNA | 487 |
| Gus_PCR_R | AGACTGCTTTTTCTTGCCGTTTTC |  |  |  |
| GUS_F | TTACGCTGAAGAGATGCTCGACTG | 57.7 | gDNA | 803 |
| 5.Bar-GusR | CTGGGCTCATTCATTGTTTGCCTCCCTG |  |  |  |
| pRB113 (Endochitinase) | |  |  |  |
| EndochitCheckF | CACTACTCCTTCCTGCACAT | 55.0 | cDNA | 437 |
| EndochitCheckR | GGTTCTTGAGGTAGGAGACC |  |  |  |
| ThEndochit_F | CTACGCCGACTACCAGAAGC | 57.4 | gDNA | 1041 |
| pUbiGFPcheckbR | ACTTTATTGCCAAATGTTTGAACGA |  |  |  |
| 113checkaF | GTCCTGCAGATGAGATCGCTCG | 61.5 | gDNA | 272 |
| Endo_PCR_R | CGTCGTCGTAGTGCTTCTGGTAGT |  |  |  |
| pUbi.GFP/pRB111 (GFP) | |  |  |  |
| GFPcheckF | GTAAACGGCCACAAGTTCAG | 55.0 | cDNA | 384 |
| GFPcheckR | TTGTCGGCGGTGATATAGAC |  |  |  |
| pUbiGFPcheck F | CAACAGCCACAAGGTCTATATCACC | 59.0 | gDNA | 210 |
| pUbiGFPcheck R | GAACTCCAGCAGGACCATGTG |  |  |  |
| GFPcheckF | GTAAACGGCCACAAGTTCAG | 56.6 | gDNA | 725 |
| pUbiGFPcheckbR | ACTTTATTGCCAAATGTTTGAACGA |  |  |  |

Primers for *GAPDH* (Glyceraldehyde 3-phosphate dehydrogenase) were synthesized on the basis of Ravel et al. (2009).

*Following PCR profile was used: initial denaturation at 95°C for 3 min followed by 40 cycles at 95°C for 30 sec, 55-61°C for 30 sec, 72°C for 45 sec, and a final extension at 72°C for 5 min.

Ravel C, Martre P, Romeuf I, Dardevet M, El-Malki R, et al. (2009) **Nucleotide polymorphism in the wheat transcriptional activator Spa influences its pattern of expression and has pleiotropic effects on grain protein composition, dough viscoelasticity, and grain hardness.** Plant Physiology 151: 2133-2144.
