# Supplementary material for: Generation of Doubled Haploid Transgenic Wheat Lines by Microspore Transformation
Source: PLoS One. 2013 Nov 18;8(11):e80155. doi: 10.1371/journal.pone.0080155 (PMC3832437; doi:10.1371/journal.pone.0080155)
Supplement: Table S4 — Effects of genotype on the percentage of green regenerants obtained from embryo-like structures formed from the transfected androgenic microspores. (DOCX) [file pone.0080155.s012.docx]

**Table S4.** Effects of genotype on the percentage of green regenerants obtained from embryo-like structures formed from the transfected androgenic microspores.

|  |  |  |  |  |
| --- | --- | --- | --- | --- |
| **Genotype** | **Market class*** | **Number of ELSs**** | **% green regenerants** | **% fertile lines^ξ^** |
| Chris | HRS | 64 | 20.3 | 7.7 |
| Express | HRS | 256 | 20.7 | 5.7 |
| Farnum | HRW | 85 | 34.1 | 82.7^ζ^ |
| Hollis | HRS | 71 | 46.5 | 78.8 |
| Louise | SWS | 75 | 29.3 | 59.1 |
| Perigee | HRS | 210 | 30.0 | None |
| WB926 | HRS | 165 | 41.2 | 22.0^ζ^ |

*HRS = hard red spring (high protein content wheat good for leavened and flat breads); HRW = hard red winter (high yielding and high protein content wheat good for leavened and flat breads); SWS = soft white spring (low protein content wheat good for baking cookies and cakes)

**Only cases were green plants regenerated from embryo-like structures (ELSs) are reported in this table

**^ξ^** Percentage of green regenerants that produced grains

^ζ^ Farnum has a tendency to produce large number of tillers whereas WB926 has a tendency to produce one or two tillers after Colchicine treatment
