# Supplementary material for: Generation of Doubled Haploid Transgenic Wheat Lines by Microspore Transformation
Source: PLoS One. 2013 Nov 18;8(11):e80155. doi: 10.1371/journal.pone.0080155 (PMC3832437; doi:10.1371/journal.pone.0080155)
Supplement: Table S5 — Effect on androgenesis of Chris microspores by concentration of A. tumefaciens in the medium for 24 hrs before filtration and addition of 200 mg·L-1 timentin. (DOCX) [file pone.0080155.s013.docx]

**Table S5.** Effect on androgenesis of Chris microspores by concentration of *A. tumefaciens* in the medium for 24 hrs before filtration and addition of 200 mg·L^-1^ timentin.

|  |  |  |  |  |  |  |  |
| --- | --- | --- | --- | --- | --- | --- | --- |
| **MT1 dose (%) in culture medium*^§^** | **0** | **0.1** | **1** | **5** | **10** | **25** | **50** |
| Viable microspores (%) at Day 7^†^ | 33^a^ | 11^b^ | 4^c^ | 0^d^ | 0^d^ | 0^d^ | 0^d^ |
| No. of embryoids at day 30 | 860^a^ | 300^b^ | 88^c^ | 0^d^ | 0^d^ | 0^d^ | 0^d^ |

^†^Means followed by the same letter in the same row were not significantly different with ANOVA and 5% LSD analysis.

*****NPB-99 (table S1)

**^§^** Culture medium containing transformed AGL-1 cells (at OD_600_ = 1.0~1.5) constitutes 0.1 to 50% of the volume of microspore co-cultivation medium (i.e., NPB-99 in this case).
