# Supplementary material for: Generation of Doubled Haploid Transgenic Wheat Lines by Microspore Transformation
Source: PLoS One. 2013 Nov 18;8(11):e80155. doi: 10.1371/journal.pone.0080155 (PMC3832437; doi:10.1371/journal.pone.0080155)
Supplement: Table S6 — Effect on androgenesis of Chris microspores by co-cultivation with 20% MT1 for varying duration (minutes) before filtration and addition of 200 mg·L-1 timentin. (DOCX) [file pone.0080155.s014.docx]

**Table S6.** Effect on androgenesis of Chris microspores by co-cultivation with 20% MT1 for varying duration (minutes) before filtration and addition of 200 mg·L^-1^ timentin.

|  |  |  |  |  |  |  |  |  |
| --- | --- | --- | --- | --- | --- | --- | --- | --- |
| **Duration (minute)**^†^ | **0** | **15** | **30** | **45** | **60** | **180** | **300** | **420** |
| Viable microspores (%)  at day 7 | 30 | 10 | 8 | 5 | 4 | 2 | 1 | 0 |
| Viable microspores (%)  at day 14^‡^ | 20^a^ | 7^b^ | 5^b^ | 4^b^ | 0^c^ | 0^c^ | 0^c^ | 0^c^ |
| No. of embryoids  at day 40 | 940^a^ | 410^b^ | 300^b^ | 282^b^ | 0^c^ | 0^c^ | 0^c^ | 0^c^ |

† 20% of *A. tumefaciens*-containing solution was added to microspore culture plate of

genotype Chris for 0 to 420 min before *A. tumefaciens* was filtered out. Timentin at the

concentration of 200 mg/L was added in the medium post co-cultivation.

‡ Means followed by the same letter in the same row were not significantly different with

ANOVA and 5% LSD analysis.
