# Supplementary material for: Generation of Doubled Haploid Transgenic Wheat Lines by Microspore Transformation
Source: PLoS One. 2013 Nov 18;8(11):e80155. doi: 10.1371/journal.pone.0080155 (PMC3832437; doi:10.1371/journal.pone.0080155)
Supplement: Table S7 — Effect of timentin in the culture medium on Chris microspore androgenesis. (DOCX) [file pone.0080155.s015.docx]

**Table S7.** Effect of timentin in the culture medium on Chris microspore androgenesis.

|  |  |  |  |  |  |  |  |  |  |
| --- | --- | --- | --- | --- | --- | --- | --- | --- | --- |
| **Timentin (mg·L^-1^)**^†^ | **0** | **100** | **200** | **300** | **400** | **500** | **600** | **700** | **800** |
| No. of embryoids at day 30^‡^ | 312^a^ | 220^b^ | 164^b^ | 156^bc^ | 144^c^ | 100^cd^ | 52^de^ | 24^e^ | 4^e^ |
| No. of embryoids transferred | 60 | 60 | 60 | 60 | 60 | 60 | 50 | 20 | 0 |
| No. of green plants germinated | 27 | 22 | 20 | 25 | 26 | 21 | 16 | 1 | 0 |
| No. of albino plants germinated | 0 | 0 | 0 | 0 | 0 | 0 | 0 | 0 | 0 |
| Plant regeneration, % | 45^a^ | 37^a^ | 33^a^ | 42^a^ | 43^a^ | 35^a^ | 32^a^ | 5^b^ | 0^b^ |

† Timentin at the concentrations of 0 to 800 mg/L was added in the embryoid induction

medium at the beginning of microspore culture.

‡ Means followed by the same letter in the same row are not significantly different with

ANOVA and 5% LSD analysis.
