# Supplementary material for: Generation of Doubled Haploid Transgenic Wheat Lines by Microspore Transformation
Source: PLoS One. 2013 Nov 18;8(11):e80155. doi: 10.1371/journal.pone.0080155 (PMC3832437; doi:10.1371/journal.pone.0080155)
Supplement: Table S8 — Effect of increasing concentrations of bialaphos used in the regeneration medium on the wild type Chris embryoids. (DOCX) [file pone.0080155.s016.docx]

**Table S8.** Effect of bialaphos in the regeneration medium on plant regeneration.

|  |  |  |  |  |  |  |
| --- | --- | --- | --- | --- | --- | --- |
| **Bialaphos, mg·L^-1^**^†^ | **0** | **1** | **2** | **4** | **6** | **8** |
| Morphology of plant at day 7^‡^ | G, green, 5cm | G, green, 3cm | G, pale green,  1cm | G, yellow, 0.5cm | NG | NG |
| No. of plants at day 28^§^ | 43^a^ | 6^b^ | 3^b^ | 2^b^ | 0^c^ | 0^c^ |
| Morphology of plants at day 28 | Green shoot with root | Green shoot with root | Pale-green shoot without root | Yellow shoot  without root | – | – |

† Embryoids of 1-2 mm in diameter derived from microspores of wild type genotype Chris

were transferred onto 190-2 medium containing bialaphos at concentrations of 0 to 8 mg/L.

‡ G=germinating, NG=non-germinating.

§ Means followed by the same letter in the same row are not significantly different with

ANOVA and 5% LSD analysis.
