# Supplementary material for: Generation of Doubled Haploid Transgenic Wheat Lines by Microspore Transformation
Source: PLoS One. 2013 Nov 18;8(11):e80155. doi: 10.1371/journal.pone.0080155 (PMC3832437; doi:10.1371/journal.pone.0080155)
Supplement: Table S9 — Staggered selection with bialaphos of wild type NPBCT embryoids for plant regeneration on 190-2 medium with different bialaphos concentrations. (DOCX) [file pone.0080155.s017.docx]

**Table S9.** Staggered selection with bialaphos of wild type NPBCT embryoids for plant regeneration on 190-2 medium with different bialaphos concentrations.

|  |  |  |  |
| --- | --- | --- | --- |
| **Plate Code of medium**^†^ | **A** | **B** | **C** |
| Bialaphos dose, mg**·**L^-1^ | 0 | 1 | 4 |
| No. of 1-2mm embryoids transferred | 80 | 80 | 80 |
| No. of green plants germinated at day 7 | 51 | 0 | 0 |
| No. of green plants germinated at day 21 | – | 2* | 0 |
| Plant regeneration (%)**^‡^** | 64^a^ | 3^b^ | 0^c^ |
| Plants transferred from A to B & C^§^ | | | |
| No. of green plants transferred from A to B & C at day 7 |  | 26 | 25 |
| No. of green plants survived at day 21 |  | 10 | 0 |
| Plant surviving (%) |  | 38^a^ | 0^b^ |
| Plants transferred from B to C^§^ | | | |
| No. of green plants transferred from B to C at day 21 |  |  | 10 |
| No. of green plant survived at day 35 |  |  | 7 |
| Plants surviving (%) |  |  | 70 |

† Embryoids of 1-2 mm in diameter derived from microspores of wild type genotype NPBCT were transferred onto 190-2 medium containing bialaphos concentrations of 0 (Plate A), 1 (Plate B), and 4 mg/L (Plate C).

‡ Means followed by the same letter in the same row are not significantly different with

ANOVA and 5% LSD analysis.

§ The 7-day-old germinated green plants on Plate A were transferred to Plate B and Plate C. Green plants survived on Plate B were later transferred to Plate C.

*Plants died 14 days after transfer to Plate C.
