# Supplementary material for: Generation of Doubled Haploid Transgenic Wheat Lines by Microspore Transformation
Source: PLoS One. 2013 Nov 18;8(11):e80155. doi: 10.1371/journal.pone.0080155 (PMC3832437; doi:10.1371/journal.pone.0080155)
Supplement: Table S10 — Staggered selection with bialaphos of Chris and WED202-16-2 embryoids derived from transformed microspores for plant regeneration on 190-2 medium with different bialaphos concentrations. (DOCX) [file pone.0080155.s018.docx]

**Table S10.** Staggered selection with bialaphos of Chris and WED202-16-2 embryoids derived from transformed microspores for plant regeneration on 190-2 medium with different bialaphos concentrations.

|  |  |  |  |
| --- | --- | --- | --- |
| **Plate Code of medium** | **A** | **B** | **C** |
| Bialaphos dose, mg**·**L^-1^ | 0 | 2 | 4 |
| Hard red spring wheat cultivar Chris | | | |
| No. of 1-2mm embryoids transferred |  | 300 |  |
| No. of green plants germinated at day 7 |  | 120 |  |
| Plant regeneration (%) |  | 40 |  |
| Plants transferred from B to C | | | |
| No. of green plants transferred from B to C at day 7 |  |  | 120 |
| No. of green plant survived at day 35* |  |  | 28 |
| Plants surviving (%) |  |  | 23 |
| Hard white spring wheat cultivar WED202-16-2 | | | |
| No. of 1-2mm embryoids transferred | 122 |  | 150 |
| No. of green plants germinated at day 7 | 85 |  | 4 |
| Plant regeneration (%) | 70 |  | 3 |
| Plants transferred from A to C |  |  |  |
| No. of green plants transferred from A to C at day 7 |  |  | 85 |
| No. of green plant survived at day 35* |  |  | 6 |
| Plants surviving (%) |  |  | 7 |

* Plants survived selection with 4 mg L^-1^ bialaphos were transplanted to greenhouse.
