# Supplementary material for: Generation of Doubled Haploid Transgenic Wheat Lines by Microspore Transformation
Source: PLoS One. 2013 Nov 18;8(11):e80155. doi: 10.1371/journal.pone.0080155 (PMC3832437; doi:10.1371/journal.pone.0080155)
Supplement: Table S11 — Xylanase activity in 200 mg of flour from wild type and transgenic T2 wheat grains. Each assay was replicated thrice. (DOCX) [file pone.0080155.s019.docx]

**Table S11.** Xylanase activity in 200 mg of flour from wild type and transgenic T_2_ wheat grains. Each assay was replicated thrice.

|  |  |  |  |
| --- | --- | --- | --- |
| **Genotype** | **Wheat cultivar** | **A_590_** | **Xylanase amount**  **μg · 60 mg^-1^** |
| Wild type | WED202-16-2 | 0.1437 | 0.0988 |
| Wild type | Chris | 0.1648 | 0.1148 |
| MT1-B4 | WED202-16-2 | 0.2989^*^ | 0.2167^*^ |
| MT1-1 | WED202-16-2 | 0.3036^*^ | 0.2203^*^ |
| MT1-6 | Chris | 0.2714^†^ | 0.1958^†^ |
| MT1-8 | Chris | 0.2789^†^ | 0.2015^†^ |
| MT1-10 | WED202-16-2 | 0.2981^*^ | 0.2161^*^ |
| MT1-11 | WED202-16-2 | 0.3241^*^ | 0.2358^*^ |
| P-value |  | 0.0108 | 0.0078 |
| LSD (0.01) |  | 0.1464 | 0.1069 |
| LSD (0.05) |  | 0.1038 | 0.0758 |

*Value is significantly different from the same wild type control with ANOVA and 1% LSD analysis. ^†^Value is significantly different from the same wild type control with ANOVA and 5% LSD analysis.
